# An Unscented Kalman Filter Based on the Adams–Bashforth Method with Applications to the State Estimation of Osprey-Type Drones Composed of Tiltable Rotor Mechanisms

**DOI:** 10.3390/s26062009

**Published:** 2026-03-23

**Authors:** Keigo Watanabe, Soma Takeda, Isaku Nagai

**Affiliations:** 1 Graduate School of Environmental, Life, Natural Science and Technology, Okayama University, Okayama 700-8530, Japan; 2 Faculty of International Economics and Management, International Pacific University (IPU), Tokyo Campus, Chiba 272-001, Japan; 3 Sony Group Corporation, Tokyo 108-0075, Japan; 4 National Institute of Technology, Mitsue College, Matsue 690-0865, Japan; nagai.isaku@matsue-ct.ac.jp

**Keywords:** unscented Kalman filter, Runge–Kutta method, Adams–Bashforth method, discretization of continuous-time models, UAV state estimation

## Abstract

In the state estimation problem for nonlinear systems, the Unscented Kalman Filter (UKF) has gained attention as an algorithm capable of accurate state estimation based on high-fidelity discretization for strongly nonlinear systems. Furthermore, for applying the UKF to continuous-time state–space models, a method employing the Runge–Kutta method in the time-update equation for sigma points has already been proposed to achieve high-precision state estimation. While this method uses high-order numerical approximations, the associated decrease in computational efficiency due to processing time becomes problematic. It is thus unsuitable for the state estimation of relatively fast-moving objects, such as autonomous vehicles and drones, which require high sampling frequencies. In this study, to reduce computational load while achieving relatively high estimation accuracy, we newly apply the Adams–Bashforth method to the UKF algorithm. The effectiveness of the proposed method is demonstrated by first explaining a low-dimensional model’s state estimation problem, followed by a comparison of estimation accuracy and computation time in state estimation simulations for the UAV model of an Osprey-type drone.

## 1. Introduction

The navigation problem for moving objects is a fundamental challenge with important engineering applications [1]. The problem of position estimation is inherently formulated as a nonlinear filtering problem, and the theory of optimal filtering has been established by researchers such as [2]. However, in practice, only the linear Kalman filter (KF) proposed by Kalman [3] and its nonlinear extensions are feasible for implementation. This is because optimal nonlinear filters are computationally too expensive to implement.

The motion models of many real-world systems, particularly autonomous mobile robots, are described in continuous time. However, since observation information from sensors is obtained discretely through sampling, the observation model becomes discrete time. Such a system is called a continuous-discrete (CD) system [4]. While the approach that directly applies the Kalman filter to this CD system is referred to as CD-KF, in practice, it is common to discretize or numerically integrate the continuous-time motion model and construct a discrete-time Kalman filter. A Kalman filter in which both the motion model and the observation model are discrete-time is called a discrete–discrete Kalman filter (DD-KF). For nonlinear dynamics or nonlinear observation systems, the CD-EKF (continuous-discrete extended Kalman filter) or DD-EKF (discrete–discrete extended Kalman filter), which apply the Kalman filter to their linearly approximated systems, are widely used [5,6,7,8]. Furthermore, as a stable algorithm that does not require linear approximation via Jacobians, the Unscented Kalman Filter (UKF) has been proposed for DD nonlinear systems [9,10,11]. This will be referred to as DD-UKF hereafter. The UKF employs a nonlinear transformation called the Unscented Transform (UT). For a system of order *n*, it generates (2n+1) sample points called sigma points, applies a nonlinear transformation to each, and then calculates the conditional expectation of the state vector and the covariance matrix by taking the sample average of the transformed points. By using this UT, the UKF enables state estimation based on high-fidelity discretization.

Since the proposal of the UKF, many researchers have advanced its development and improvement. Särkkä [12] derived continuous-time and continuous-discrete versions of the UKF and applied them to nonlinear continuous-time filtering and re-entry vehicle tracking problems. van der Merwe [13] proposed the square-root UKF to improve numerical stability, and Ito and Xiong [14] analyzed the convergence of Gaussian filters, including the UKF. Julier [15] proposed the spherical simplex UT to reduce the number of sigma points. Meanwhile, Haykin [16] advanced research on the integration of neural networks and Kalman filtering, demonstrating new possibilities for nonlinear adaptive filtering. Elliott [17] contributed to the foundations of filtering theory through the theory of hidden Markov models and stochastic control. Recently, Singh [18] comprehensively reviewed the development of Gaussian filtering, including the UKF. In particular, recent studies have combined the UKF with outlier handling and event-triggered mechanisms. For example, Ref. [19] proposed a dual-event-triggering ANFIS-based UKF for cluster cooperative navigation with measurement anomalies, demonstrating potential applications to navigation problems in complex environments.

From the perspective of numerical integration methods, Takeno and Katayama [20] applied the Runge–Kutta (RK) method to the time-update equations for sigma points and demonstrated that high-precision state estimation is possible for several models. Furthermore, Takeno and Katayama [21] applied Heun’s method, an improved version of the Euler method, to the prediction step. Kulikov and Kulikova [22,23] have advanced research on high-precision numerical implementation of the continuous-discrete UKF. However, discussions on these UKFs have focused primarily on estimation accuracy, with little consideration given to computational efficiency. Due to its characteristic of performing nonlinear transformations on each of the (2n+1) sigma points in the UT algorithm, the UKF has the drawback of increased computation time as the system order *n* grows. For state estimation of large-scale real-world models, both accuracy and real-time performance are required, making the improvement of computational efficiency in UKF algorithms critically important [24]. In recent years, research addressing this computational efficiency challenge has begun to emerge. Wang et al. [25] proposed an adaptive step-size UKF based on the degree of nonlinearity, achieving a balance between accuracy and computational cost by dynamically adjusting the step size in highly nonlinear regions. Researchers in the aerospace domain [26] introduced a high-efficiency UKF utilizing parallel computation for sigma point propagation, demonstrating significant speedup for multi-target trajectory estimation. The integration of machine learning techniques with UKF has also been explored; a GAN-enhanced UKF framework [27] dynamically predicts and updates filter parameters in real-time, improving estimation accuracy without sacrificing computational performance. In the realm of numerical implementation, Kulikova and Kulikov [28] developed square-root information-type methods for continuous-discrete extended Kalman filtering, enhancing numerical stability which is crucial for efficient computation. Furthermore, theoretical advances in sensor scheduling for continuous-discrete systems [29] provide insights into optimizing the trade-off between resource allocation and estimation accuracy. A comparative evaluation of nonlinear filters [30] further contextualizes the performance of the UKF against other methods in practical tracking applications. These recent developments underscore the growing recognition of computational efficiency as a critical factor in the practical deployment of nonlinear Kalman filters.

Regarding Unmanned Aerial Vehicle (UAV) navigation, comprehensive reviews exist on topics such as autonomous navigation in GPS-denied environments [31], UAV positioning using GNSS [32], and sensor-based autonomous localization [33]. These reviews highlight the importance of filtering techniques in UAV navigation. Therefore, in this study, to improve the computational efficiency of the UKF, we propose a UKF that applies the Adams–Bashforth (AB) method, instead of the RK method, to the time-update equations for sigma points. The RK method is a single-step numerical integration method; it calculates the next numerical point $\left(x\left(t_{k+1}\right),t_{k+1}\right)$ based solely on the current point $x(t_{k})$. Furthermore, when the order of accuracy is s (s<5), *s* function evaluations are required to generate the next numerical point. On the other hand, the AB method is a multi-step numerical integration method. For an accuracy order of 4, the next numerical point $x(t_{k+1})$ is calculated based on the past four points $x\left(t_{k-3}\right),x\left(t_{k-2}\right),x\left(t_{k-1}\right),x\left(t_{k}\right)$, and only one function evaluation is needed to generate the next numerical point. Thus, regardless of the accuracy order *s* of the AB method, the number of function evaluations is always one. Therefore, compared to the fixed-step RK method, computation with the AB method is more efficient.

It should be noted, as a related study focusing on the degradation of computational efficiency of the RK method when applying numerical integration to nonlinear Kalman filters, that He et al. [34] have already conducted research applying a multi-step method based on the Adams–Bashforth–Moulton (ABM) method to the EKF. The objective of this study is to demonstrate that the UKF applying the AB method to the time-update equations for sigma points in the UT can achieve estimation with computational efficiency superior to that of the RK method, while maintaining comparable estimation accuracy. In addition to comparison with the RK method, we also compare the differences in estimation accuracy and computation time resulting from varying orders (2nd to 6th) of the AB method itself.

The effectiveness of the proposed method is demonstrated through state estimation in a MATLAB 2022a simulation environment using two nonlinear models. Specifically, as a preliminary experiment, we first apply the method to a low-dimensional falling object model used in previous research and compare estimation performance under identical conditions. Subsequently, as an application to a more complex model, we perform a similar comparison of estimation performance using a UAV model of an Osprey-type drone.

The structure of this paper is as follows. Section 2 formulates the continuous-discrete system and the state estimation problem. Section 3 discusses the integration of the UT with the Runge–Kutta method and the Adams–Bashforth method, presenting the time-update equations for sigma points based on each method. Section 4 details the modeling of the Osprey-type drone. After defining the coordinate systems, the rotational and translational motions are explained in detail. Section 5 describes the control law and control allocation. Specifically, a controller based on the computed torque method and the control input allocation problem are detailed. Section 6, as a preliminary experiment, applies the sigma-point time-update equations presented in Section 3 to the falling object model used in previous research, comparing estimation accuracy and computation time. Section 7 first derives the state equations and sigma-point time-update equations for the Osprey-type UAV model, then performs comparisons of estimation accuracy and computation time similar to those in Section 6. Furthermore, we provide discussions based on a broader range of estimation results, including not only comparisons with the RK method but also differences in estimation performance due to the order of the AB method and comparisons of estimation performance under different sampling periods. Section 6 summarizes the paper and presents concluding remarks.

## 2. Continuous-Discrete System and Problem Formulation

### 2.1. Continuous-Discrete System

The motion of many real-world systems, particularly moving objects such as UAVs, is described in continuous time, while observations from sensors are obtained discretely through sampling. Such systems are called CD systems, and the foundations of their filtering theory are detailed in Jazwinski [4].

In this study, we consider a system described by the following nonlinear continuous-time state equation and discrete-time observation equation:

(1) $$\begin{array}{cc} \dot{x}\left(t\right) & =f_{c}\left(x\left(t\right),u\left(t\right)\right)+w_{c}\left(t\right), \end{array}$$

(2) $$\begin{array}{cc} y(t_{k}) & =h_{m}\left(x\left(t_{k}\right)\right)+v\left(t_{k}\right). \end{array}$$

Here, *t* denotes continuous time, $t_{k}=k\Delta t$ (k=0,1,…) denotes sampling instants, and Δt is the sampling interval. $x\left(t\right)\in R^{n}$ is the state vector, $u\left(t\right)\in R^{m}$ is the input vector, and $y\left(t_{k}\right)\in R^{p}$ is the output vector. $f_{c}:R^{n}\times R^{m}\to R^{n}$ and $h_{m}:R^{n}\to R^{p}$ are nonlinear functions. Furthermore, $w_{c}\left(t\right)\in R^{n}$ is the system noise, and $v\left(t_{k}\right)\in R^{p}$ is the observation noise, both assumed to be Gaussian white noises with zero mean and covariance matrices $Q_{c}$ and *R*, respectively.

### 2.2. Discrete–Discrete Unscented Kalman Filter

Discretizing the continuous-time state Equation (1) using some numerical integration method yields the following discrete-time state equation:

(3) $$\begin{array}{c} x\left(t_{k+1}\right)=f_{d}\left(x\left(t_{k}\right),u\left(t_{k}\right)\right)+w\left(t_{k}\right) \end{array}$$

where $f_{d}$ is an approximation function obtained through the numerical integration method, and $w(t_{k})$ is the system noise arising from discretization. The UKF can be applied to the DD system composed of Equations (2) and (3).

The UKF is an algorithm that performs state estimation for nonlinear systems using the Unscented Transform (UT) [9,10,11]. Hereafter, the filtered estimate is denoted as $\hat{x}\left(t_{k}|t_{k}\right)$, its covariance matrix as $P(t_{k}|t_{k})$, the one-step predicted estimate as $\hat{x}\left(t_{k+1}|t_{k}\right)$, and its covariance matrix as $P(t_{k+1}|t_{k})$. The initial estimate $\hat{x}\left(0|0\right)$ and initial covariance matrix P(0|0) are assumed to be known.

The UKF algorithm consists of a prediction step and an update step. The detailed derivation and specific equations are provided in Appendix A; here, we present only an overview.

**Prediction Step**: From the estimate $\hat{x}\left(t_{k}|t_{k}\right)$ and covariance matrix $P(t_{k}|t_{k})$ at time $t_{k}$, (2n+1) sigma points $X_{i}\left(t_{k}|t_{k}\right)$ and corresponding weights $W_{i}$ are generated. These sigma points are propagated through the nonlinear function $f_{d}$, and the one-step predicted estimate $\hat{x}\left(t_{k+1}|t_{k}\right)$ and its covariance matrix $P(t_{k+1}|t_{k})$ are computed by taking the weighted average.

**Update Step**: The predicted sigma points $X_{i}\left(t_{k+1}|t_{k}\right)$ are substituted into the observation function $h_{m}$ to compute the predicted output $\hat{y}\left(t_{k+1}|t_{k}\right)$, its covariance $P_{yy}$, and the cross-covariance between state and output $P_{x\nu }$. The innovation $\nu (t_{k+1})$ is computed using the actual observation $y(t_{k+1})$, and the estimate $\hat{x}\left(t_{k+1}|t_{k+1}\right)$ and covariance matrix $P(t_{k+1}|t_{k+1})$ are updated via the Kalman gain $K(t_{k+1})$.

For the specific computational procedure and complete set of equations for the UKF, please refer to Appendix A.

## 3. Unscented Transform and Discretization Methods

### 3.1. Discrete-Time State–Space Model

Consider the following continuous-time nonlinear system:

(4) $$\begin{array}{cc} \frac{dx}{dt}=f_{c}\left(x\right), & \;x\left(0\right)=x_{0} \end{array}$$

where $x\in R^{n}$ is the state vector and $x_{0}$ is the initial value. Discretizing this continuous-time system using some numerical integration method yields the following time-update equation:

(5) $$\begin{array}{cc} x\left(t_{k+1}\right)=f_{d}\left(x\left(t_{k}\right)\right), & \;k=0,1,\ldots \end{array}$$

The observation equation is given similarly to Equation (2) as

(6) $$\begin{array}{cc} y\left(t_{k}\right)=h_{m}\left(x\left(t_{k}\right)\right)+v\left(t_{k}\right), & \;k=0,1,\ldots \end{array}$$

where *v* is Gaussian white noise with zero mean and covariance matrix *R*. Equations (5) and (6) constitute the discrete-time state–space model.

The objective of this study is to propose a nonlinear filtering algorithm that sequentially estimates the state vector $x(t_{k})$ based on the observation data $y(t_{k})$. Takeno and Katayama [20] demonstrated that combining the UKF with the RK method enables high-precision state estimation. In this study, we show that by combining the AB method with the UT instead of the RK method, relatively high-precision state estimation can be achieved while reducing computational load.

For the general theory of numerical solutions for stochastic differential equations, please refer to Kloeden and Platen [35].

### 3.2. Integration of UT and the Runge–Kutta Method

When applying the RK method to the time update of sigma points in the UKF, numerical integration is performed for each sigma point. The sigma point matrix $X_{k}$ at time *k* with its element $X_{i,j,k}$ is defined as follows:

(7) $$X_{k}=\left[\begin{array}{ccc} X_{1,1,k} & \ldots & X_{1,2n+1,k}\\ \vdots & \ddots & \vdots \\ X_{n,1,k} & \ldots & X_{n,2n+1,k} \end{array}\right].$$

Here, i=1,…,n denotes the element of the state vector, and j=1,…,2n+1 denotes the index of the sigma point. Let the step size be Δt=h and the time instant be $t_{k}=kh$.

When applying the 4th-order Runge–Kutta method, the time-update equation for the sigma points is expressed in the following general form:

(8) $$\begin{array}{c} X_{i,j,k+1}^{-}=X_{i,j,k}+\frac{h}{6}\left(a_{1,ij}+2a_{2,ij}+2a_{3,ij}+a_{4,ij}\right). \end{array}$$

Here, $a_{1,ij},a_{2,ij},a_{3,ij},a_{4,ij}$ are the function evaluations at each stage of the RK method. The specific definitions and detailed derivations are provided in Appendix B.

By taking the weighted average of these updated sigma points, the predicted value of the state vector (Equation (A10)) and the predicted covariance matrix (Equation (A11)) are obtained.

### 3.3. Integration of UT and the Adams–Bashforth Method

This section describes the integration of the AB method with the UT, which is the core of this study. The ordinary AB method is a multi-step method that calculates the next point using information from multiple past points. In this study, we extend and apply this method to the sigma point matrix of the UKF.

First, the result of substituting each column (each sigma point) of the sigma point matrix $X_{k}$ at time *k* into the nonlinear function $f_{c}$ is defined as the following n×(2n+1) matrix:

(9) $$F_{c}\left(X_{k}\right)=\left[\begin{array}{ccc} f_{1}\left(X_{1,1,k}\right) & \ldots & f_{1}\left(X_{1,2n+1,k}\right)\\ \vdots & \ddots & \vdots \\ f_{n}\left(X_{n,1,k}\right) & \ldots & f_{n}\left(X_{n,2n+1,k}\right) \end{array}\right].$$

This $F_{c}\left(X_{k}\right)$ is a matrix that stores the results of evaluating the nonlinear function for all sigma points at time *k*. While the ordinary AB method uses function evaluation values for past state vectors, the feature of the proposed method is that it stores function evaluation matrices for past sigma point matrices $F_{c}\left(X_{k-1}\right),F_{c}\left(X_{k-2}\right),\ldots$ and uses them as “past information.”

Below, we present the time-update equations for sigma points using the 2nd- to 6th-order AB methods [36,37].

- **2nd-order Adams–Bashforth method**:
  (10) $$\begin{array}{c} X_{k+1}^{-}=X_{k}+\frac{h}{2}\left(3F_{c}\left(X_{k}\right)-F_{c}\left(X_{k-1}\right)\right). \end{array}$$
- **3rd-order Adams–Bashforth method**:
  (11) $$\begin{array}{c} X_{k+1}^{-}=X_{k}+\frac{h}{12}\left(23F_{c}\left(X_{k}\right)-16F_{c}\left(X_{k-1}\right)+5F_{c}\left(X_{k-2}\right)\right). \end{array}$$
- **4th-order Adams–Bashforth method**:
  (12) $$\begin{array}{c} X_{k+1}^{-}=X_{k}+\frac{h}{24}\left(55F_{c}\left(X_{k}\right)-59F_{c}\left(X_{k-1}\right)+37F_{c}\left(X_{k-2}\right)-9F_{c}\left(X_{k-3}\right)\right). \end{array}$$
- **5th-order Adams–Bashforth method**:
  (13) $$\begin{array}{cc} X_{k+1}^{-}=X_{k}+\frac{h}{720}( & 1901F_{c}\left(X_{k}\right)-2774F_{c}\left(X_{k-1}\right)\\ \; & +2616F_{c}\left(X_{k-2}\right)-1274F_{c}\left(X_{k-3}\right)+251F_{c}\left(X_{k-4}\right)). \end{array}$$
- **6th-order Adams–Bashforth method**:

(14) $$\begin{array}{cc} X_{k+1}^{-}=X_{k}+\frac{h}{1440}( & 4277F_{c}\left(X_{k}\right)-7923F_{c}\left(X_{k-1}\right)\\ \; & +9982F_{c}\left(X_{k-2}\right)-7298F_{c}\left(X_{k-3}\right)\\ \; & +2877F_{c}\left(X_{k-4}\right)-475F_{c}\left(X_{k-5}\right)). \end{array}$$

Here, $X_{k}$ and $X_{k+1}^{-}$ are n×(2n+1) matrices. These equations are an extension of the ordinary AB method formulas to the sigma point matrix, representing *an original formulation for integrating the numerical integration method into the specific filtering method of UKF*. In particular, the method of storing and utilizing the results of function evaluations for past sigma point matrices is a novel aspect of this approach not found in existing research.

### 3.4. Comparison of Computational Complexity: Runge–Kutta Method vs. Adams–Bashforth Method

We compare the computational complexity of the RK and AB methods in the UT from the following two perspectives.

#### 3.4.1. Computational Complexity Comparison Based on “Number of Stages”

The difference width Δt=h in discretization is generally called the step size in numerical computation. The RK method computes the derivative $f_{i}\left(X\right)$ multiple times while advancing one step (this is referred to as the “number of stages”). For example, the 4th-order RK method requires four stages of computation, and its global truncation error is $O(h^{4})$.

On the other hand, since the AB method utilizes previously computed sigma point matrices $F_{c}\left(X_{k-1}\right),F_{c}\left(X_{k-2}\right),\ldots$, only one computation of $F_{c}\left(X_{k}\right)$ is sufficient to derive the time-update equation for the current sigma points. That is, regardless of the accuracy order of the AB method, the number of stages is always 1/4 that of the RK method.

#### 3.4.2. Computational Complexity Comparison Based on “Number of Elements in the Sigma Point Set”

Generally, when simulating large-scale models in real space, the number of state variables *n* can become very large (millions to tens of millions). In the UKF, since the size of the sigma point set is (2n+1), this effect becomes pronounced. The RK method performs four stages of derivative computation for each sigma point, for each element of the *n*-dimensional state vector and for each element of the sigma point set.

In contrast, the AB method requires only one stage of function computation for each element of the sigma point set. Furthermore, for past sigma point sets, it only involves multiplying the entire matrix by coefficients according to the accuracy order. Therefore, the impact of an increase in the number of state variables is significantly smaller compared to the RK method.

This theoretical comparison of computational complexity will be verified through numerical experiments presented later in Section 6 and Section 7.

## 4. Modeling of the Osprey-Type Drone

### 4.1. Definition of Coordinate Systems

**Figure 1:**
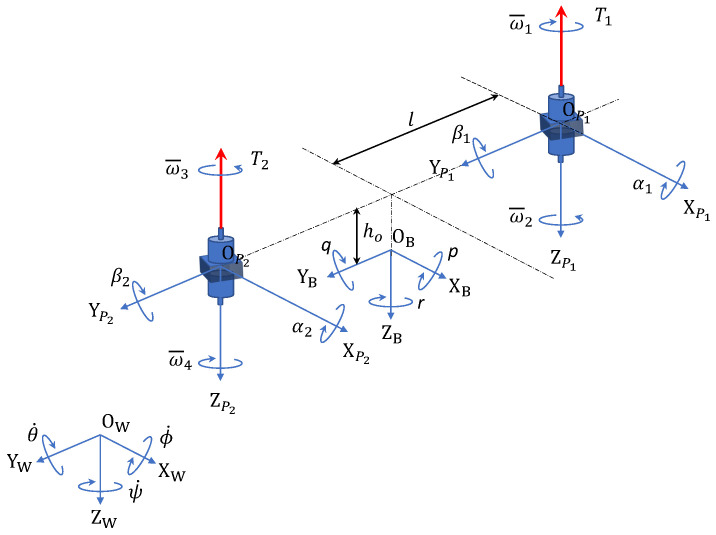
Definition of the coordinate systems of the Osprey-type drone. The red arrows represent the thrust generated by each coaxial rotor.

Figure 1 shows the coordinate systems used in this study for the Osprey-type drone. The world coordinate system $F_{W}$ is a right-handed coordinate system with origin $O_{W}$ and axes $X_{W}$, $Y_{W}$, $Z_{W}$, where $Z_{W}$ is positive vertically downward. The body coordinate system $F_{B}$ has its origin $O_{B}$ at the center of gravity of the vehicle. It is also a right-handed coordinate system with axes $X_{B}$, $Y_{B}$, $Z_{B}$ and $Z_{B}$ positive vertically downward. The positive direction of the $X_{B}$ axis is designated as the forward direction of the vehicle.

The coordinate system for the first coaxial rotor, $F_{P_{1}}$, has origin $O_{P_{1}}$ and axes $X_{P_{1}}$, $Y_{P_{1}}$, $Z_{P_{1}}$. Similarly, the coordinate system for the second coaxial rotor, $F_{P_{2}}$, has origin $O_{P_{2}}$ and axes $X_{P_{2}}$, $Y_{P_{2}}$, $Z_{P_{2}}$. The coordinate system for the *i*-th (i=1,2) coaxial rotor is integrated into $F_{P_{i}}$. In the body coordinate system $F_{B}$, the angular velocities about the $X_{B}$, $Y_{B}$, $Z_{B}$ axes are (p,q,r), respectively. Furthermore, in the rotor coordinate system $F_{P_{i}}$, the tilt angles about the $X_{P_{i}}$, $Y_{P_{i}}$ axes are $\left(\alpha_{i},\beta_{i}\right)$. The initial tilt angles $\alpha_{i}$ and $\beta_{i}$ are 0. Due to servo motor characteristics, the range of $\alpha_{i}$ is $-2/\pi \leq \alpha_{i}\leq 2/\pi$, while $\beta_{i}$ is unlimited.

Let the Euler angles in the world coordinate system be $\eta ={\left[\phi \;\theta \;\psi \right]}^{T}$. The rotation matrices about the *x*, *y*, *z* axes, $R_{x}$, $R_{y}$, $R_{z}$, can be expressed as follows (*S* denotes sin, and *C* denotes cos):

(15) $$\begin{array}{cc} R_{x} & =\left[\begin{array}{ccc} 1 & 0 & 0\\ 0 & C_{\phi } & -S_{\phi }\\ 0 & S_{\phi } & C_{\phi } \end{array}\right], \end{array}$$

(16) $$\begin{array}{cc} R_{y} & =\left[\begin{array}{ccc} C_{\theta } & 0 & S_{\theta }\\ 0 & 1 & 0\\ -S_{\theta } & 0 & C_{\theta } \end{array}\right], \end{array}$$

(17) $$\begin{array}{cc} R_{z} & =\left[\begin{array}{ccc} C_{\psi } & -S_{\psi } & 0\\ S_{\psi } & C_{\psi } & 0\\ 0 & 0 & 1 \end{array}\right]. \end{array}$$

From these $R_{x}$, $R_{y}$, $R_{z}$, the transformation matrix from the body coordinate system to the world coordinate system, RBW, can be expressed as

(18) RBW=Rz(ψ)Ry(θ)Rx(ϕ)=CθCψSϕSθCψ−CϕSψCϕSθCψ+SϕSψCθSψSϕSθSψ+CϕCψCϕSθSψ−SϕCψ−SθSϕCθCϕCθ.

Also, let the transformation matrix for angular velocity from the world coordinate system to the body coordinate system be $W_{\eta }$. Then, $\omega_{B}≜{\left[p\;q\;r\right]}^{T}$ is

(19) $$\begin{array}{cc} \omega_{B} & =W_{\eta }\dot{\eta } \end{array}$$

and its inverse is given by

(20) $$\begin{array}{cc} \dot{\eta } & =W_{\eta }^{-1}\omega_{B}. \end{array}$$

Here, it is found in [38,39] that

(21) $$\begin{array}{cc} W_{\eta } & =\left[\begin{array}{ccc} 1 & 0 & -S_{\theta }\\ 0 & C_{\phi } & C_{\theta }S_{\phi }\\ 0 & -S_{\phi } & C_{\theta }C_{\phi } \end{array}\right], \end{array}$$

(22) $$\begin{array}{cc} W_{\eta }^{-1} & =\left[\begin{array}{ccc} 1 & S_{\phi }T_{\theta } & C_{\phi }T_{\theta }\\ 0 & C_{\phi } & -S_{\phi }\\ 0 & S_{\phi }/C_{\theta } & C_{\phi }/C_{\theta } \end{array}\right] \end{array}$$

where $T_{x}=\tan\left(x\right)$. Note that $W_{\eta }$ is invertible unless θ=(2k−1)ϕ/2,(k∈Z). That is, it is invertible as long as it does not take the specific ϕ=±π/(2k−1) where gimbal lock occurs.

From the tilt angles $\alpha_{i}$ and $\beta_{i}$, where (i=1,2) in each rotor coordinate system, the transformation matrix from each rotor coordinate system to the body coordinate system, RPiB, can be expressed as

(23) RP1B=Rz(0)Ry(β1)Rx(α1)=Cβ1Sα1Sβ1Cα1Sβ10Cα1−Sα1−Sβ1Sα1Cβ1Cα1Cβ1,

(24) RP2B=Rz(0)Ry(β2)Rx(α2)=Cβ2Sα2Sβ2Cα2Sβ20Cα2−Sα2−Sβ2Sα2Cβ2Cα2Cβ2.

### 4.2. Rotational Motion

First, the angular velocity $\omega_{P_{i}}$ and angular acceleration ${\dot{\omega }}_{P_{i}}$ of the *i*-th (i=1,2) coaxial rotor can be expressed as

(25) $$\left\{\begin{array}{c} \omega_{P_{1}}={\left[\begin{array}{ccc} {\dot{\alpha }}_{1} & {\dot{\beta }}_{1} & {\bar{\omega }}_{2}-{\bar{\omega }}_{1} \end{array}\right]}^{T}\\ \omega_{P_{2}}={\left[\begin{array}{ccc} {\dot{\alpha }}_{2} & {\dot{\beta }}_{2} & {\bar{\omega }}_{3}-{\bar{\omega }}_{4} \end{array}\right]}^{T} \end{array}\right.,$$

(26) $$\left\{\begin{array}{c} {\dot{\omega }}_{P_{1}}={\left[\begin{array}{ccc} {\ddot{\alpha }}_{1} & {\ddot{\beta }}_{1} & {\dot{\bar{\omega }}}_{2}-{\dot{\bar{\omega }}}_{1} \end{array}\right]}^{T}\\ {\dot{\omega }}_{P_{2}}={\left[\begin{array}{ccc} {\ddot{\alpha }}_{2} & {\ddot{\beta }}_{2} & {\dot{\bar{\omega }}}_{3}-{\dot{\bar{\omega }}}_{4} \end{array}\right]}^{T} \end{array}\right.$$

where ${\bar{\omega }}_{1}$, ${\bar{\omega }}_{2}$ are the angular velocities of each brushless motor in the first coaxial rotor, and ${\bar{\omega }}_{3}$, ${\bar{\omega }}_{4}$ are those of the second coaxial rotor.

Next, the reaction torque $\tau_{c,i}$ of the *i*-th (i=1,2) coaxial rotor can be expressed as

(27) $$\left\{\begin{array}{c} \tau_{c,1}={\left[\begin{array}{ccc} 0 & 0 & k_{t}\left({\bar{\omega_{2}}}^{2}-{\bar{\omega }}_{1}^{2}\right) \end{array}\right]}^{T}\\ \tau_{c,2}={\left[\begin{array}{ccc} 0 & 0 & k_{t}\left({\bar{\omega_{3}}}^{2}-{\bar{\omega }}_{4}^{2}\right) \end{array}\right]}^{T} \end{array}\right.$$

where $k_{t}>0$ represents the torque coefficient.

Also, the thrust $T_{i}$ of the *i*-th (i=1,2) coaxial rotor can be expressed as

(28) $$\left\{\begin{array}{c} T_{1}={\left[\begin{array}{ccc} 0 & 0 & -T_{1} \end{array}\right]}^{T}={\left[\begin{array}{ccc} 0 & 0 & -k_{f}\left({\bar{\omega }}_{1}^{2}+{\bar{\omega }}_{2}^{2}\right) \end{array}\right]}^{T}\\ T_{2}={\left[\begin{array}{ccc} 0 & 0 & -T_{2} \end{array}\right]}^{T}={\left[\begin{array}{ccc} 0 & 0 & -k_{f}\left({\bar{\omega }}_{3}^{2}+{\bar{\omega }}_{4}^{2}\right) \end{array}\right]}^{T} \end{array}\right.$$

where $k_{f}>0$ represents the thrust coefficient of the coaxial rotor.

Using the Newton–Euler method, the torque $\tau_{P_{i}}$ generated by the coaxial rotor can be expressed as

(29) $$\tau_{P_{i}}=I_{P}{\dot{\omega }}_{P_{i}}+\omega_{P_{i}}\times I_{P}\omega_{P_{i}}+\tau_{c,i}.$$

Here, since the angular velocity and angular acceleration caused by the servo motor tilt are instantaneous and minute, they can be expressed as

(30) $$\begin{array}{c} {\dot{\alpha }}_{i}=0,\;{\ddot{\alpha }}_{i}=0,\;{\dot{\beta }}_{i}=0,\;{\ddot{\beta }}_{i}=0\;\left(i=1,2\right);\;{\dot{\omega }}_{P_{j}}=0\;\left(j=1,2,3,4\right). \end{array}$$

Therefore, $\omega_{P_{i}}$ and $\tau_{P_{i}}$ become as follows:

(31) $$\left\{\begin{array}{c} \omega_{P_{1}}={\left[\begin{array}{ccc} 0 & 0 & \bar{\omega_{2}}-{\bar{\omega }}_{1} \end{array}\right]}^{T}\\ \omega_{P_{2}}={\left[\begin{array}{ccc} 0 & 0 & \bar{\omega_{3}}-{\bar{\omega }}_{4} \end{array}\right]}^{T} \end{array}\right.,$$

(32) $$\left\{\begin{array}{c} \tau_{P_{1}}={\left[\begin{array}{ccc} 0 & 0 & k_{t}\left({\bar{\omega }}_{2}^{2}-{\bar{\omega }}_{1}^{2}\right) \end{array}\right]}^{T}\\ \tau_{P_{2}}={\left[\begin{array}{ccc} 0 & 0 & k_{t}\left({\bar{\omega }}_{3}^{2}-{\bar{\omega }}_{4}^{2}\right) \end{array}\right]}^{T} \end{array}\right..$$

#### 4.2.1. Rotational Torque $\tau_{B}$ Generated by Rotor Thrust

The rotational torque $\tau_{B}$ generated on the vehicle by rotor thrust can be expressed using the position vector OriB of each rotor in the body coordinate system as follows:

(33) τB=∑i=12(OPiB×RPiBTi),

(34) OPiB=0(−1)il−hoT.

The calculation details for i=1,2 are shown below:

RP1BT1=Cβ1Sα1Sβ1Cα1Sβ10Cα1−Sα1−Sβ1Sα1Cβ1Cα1Cβ100−T1=−Cα1Sβ1T1Sα1T1−Cα1Cβ1T1,Rr2BT2=Cβ2Sα2Sβ2Cα2Sβ20Cα2−Sα2−Sβ2Sα2Cβ2Cα2Cβ200−T2=−Cα2Sβ2T2Sα2T2−Cα2Cβ2T2,

OP1B×RP1BT1=0−l−ho×−Cα1Sβ1T1Sα1T1−Cα1Cβ1T1=lCα1Cβ1T1+hoSα1T1hoCα1Sβ1T1−lCα1Sβ1T1,OP2B×RP2BT2=0l−ho×−Cα2Sβ2T2Sα2T2−Cα2Cβ2T2=−lCα2Cβ2T2+hoSα2T2hoCα2Sβ2T2lCα2Sβ2T2.

Therefore, $\tau_{B}={\left[\tau_{x}^{B}\;\tau_{y}^{B}\;\tau_{z}^{B}\right]}^{T}$ can be expressed as

(35) $$\begin{array}{cc} \tau_{B}=\left[\begin{array}{c} \tau_{x}^{B}\\ \tau_{y}^{B}\\ \tau_{z}^{B} \end{array}\right]\; & =\left[\begin{array}{c} \left(lC_{\alpha_{1}}C_{\beta_{1}}+h_{o}S_{\alpha_{1}}\right)T_{1}-\left(lC_{\alpha_{2}}C_{\beta_{2}}-h_{o}S_{\alpha_{2}}\right)T_{2}\\ h_{o}C_{\alpha_{1}}S_{\beta_{1}}T_{1}+h_{o}C_{\alpha_{2}}S_{\beta_{2}}T_{2}\\ -lC_{\alpha_{1}}S_{\beta_{1}}T_{1}+lC_{\alpha_{2}}S_{\beta_{2}}T_{2} \end{array}\right]\\ \; & =k_{f}\left[\begin{array}{c} \begin{array}{cc} \; & \left(lC_{\alpha_{1}}C_{\beta_{1}}+h_{o}S_{\alpha_{1}}\right)\left({\bar{\omega }}_{1}^{2}+{\bar{\omega }}_{2}^{2}\right)\\ \; & \;\;-\left(lC_{\alpha_{2}}C_{\beta_{2}}-h_{o}S_{\alpha_{2}}\right)\left({\bar{\omega }}_{3}^{2}+{\bar{\omega }}_{4}^{2}\right)\\ \; & h_{o}C_{\alpha_{1}}S_{\beta_{1}}\left({\bar{\omega }}_{1}^{2}+{\bar{\omega }}_{2}^{2}\right)+h_{o}C_{\alpha_{2}}S_{\beta_{2}}\left({\bar{\omega }}_{3}^{2}+{\bar{\omega }}_{4}^{2}\right)\\ \; & -lC_{\alpha_{1}}S_{\beta_{1}}\left({\bar{\omega }}_{1}^{2}+{\bar{\omega }}_{2}^{2}\right)+lC_{\alpha_{2}}S_{\beta_{2}}\left({\bar{\omega }}_{3}^{2}+{\bar{\omega }}_{4}^{2}\right) \end{array} \end{array}\right]. \end{array}$$

#### 4.2.2. Reaction Torque $\tau_{c}$ Generated by Rotor Rotation

The reaction torque $\tau_{c}$ generated by rotor rotation can be expressed using the reaction torque $\tau_{P_{i}}$ of each rotor as follows:

(36) τc=∑i=12(RPiBτPi).

The calculation details for i=1,2 are shown below:

RP1BτP1=Cβ1Sα1Sβ1Cα1Sβ10Cα1−Sα1−Sβ1Sα1Cβ1Cα1Cβ100kt(ω¯22−ω¯12)=ktCα1Sβ1(ω¯22−ω¯12)−Sα1(ω¯22−ω¯12)Cα1Cβ1(ω¯22−ω¯12),

RP2BτP2=Cβ2Sα2Sβ2Cα2Sβ20Cα2−Sα2−Sβ2Sα2Cβ2Cα2Cβ200kt(ω¯32−ω¯42)=ktCα2Sβ2(ω¯32−ω¯42)−Sα2(ω¯32−ω¯42)Cα2Cβ2(ω¯32−ω¯42).

Therefore, $\tau_{c}={\left[\tau_{x}^{c}\;\tau_{y}^{c}\;\tau_{z}^{c}\right]}^{T}$ can be expressed as

(37) $$\begin{array}{cc} \tau_{c} & =\left[\begin{array}{c} \tau_{x}^{c}\\ \tau_{y}^{c}\\ \tau_{z}^{c} \end{array}\right]\\ \; & =k_{t}\left[\begin{array}{c} C_{\alpha_{1}}S_{\beta_{1}}\left({\bar{\omega }}_{2}^{2}-{\bar{\omega }}_{1}^{2}\right)+C_{\alpha_{2}}S_{\beta_{2}}\left({\bar{\omega }}_{3}^{2}-{\bar{\omega }}_{4}^{2}\right)\\ -S_{\alpha_{1}}\left({\bar{\omega }}_{2}^{2}-{\bar{\omega }}_{1}^{2}\right)-S_{\alpha_{2}}\left({\bar{\omega }}_{3}^{2}-{\bar{\omega }}_{4}^{2}\right)\\ C_{\alpha_{1}}C_{\beta_{1}}\left({\bar{\omega }}_{2}^{2}-{\bar{\omega }}_{1}^{2}\right)+C_{\alpha_{2}}C_{\beta_{2}}\left({\bar{\omega }}_{3}^{2}-{\bar{\omega }}_{4}^{2}\right) \end{array}\right]. \end{array}$$

#### 4.2.3. Vehicle Coriolis Force $\tau_{cori}$

The vehicle Coriolis force $\tau_{cori}$ can be expressed using the vehicle’s angular velocity $\omega_{B}$ and its inertia matrix $I_{B}$ as follows:

(38) $$\tau_{cori}=\omega_{B}\times I_{B}\omega_{B}.$$

Here, since

$$\begin{array}{c} I_{B}=\left[\begin{array}{ccc} I_{Bxx} & 0 & 0\\ 0 & I_{Byy} & 0\\ 0 & 0 & I_{Bzz} \end{array}\right],\;\omega_{B}=\left[\begin{array}{c} p\\ q\\ r \end{array}\right] \end{array}$$

we have

$$I_{B}\omega_{B}=\left[\begin{array}{ccc} I_{Bxx} & 0 & 0\\ 0 & I_{Byy} & 0\\ 0 & 0 & I_{Bzz} \end{array}\right]\left[\begin{array}{c} p\\ q\\ r \end{array}\right]=\left[\begin{array}{c} pI_{Bxx}\\ qI_{Byy}\\ rI_{Bzz} \end{array}\right].$$

Therefore, letting $\tau_{cori}={\left[\tau_{x}^{cori}\;\tau_{y}^{cori}\;\tau_{z}^{cori}\right]}^{T}$, we obtain

(39) $$\begin{array}{cc} \tau_{cori}=\left[\begin{array}{c} \tau_{x}^{cori}\\ \tau_{y}^{cori}\\ \tau_{z}^{cori} \end{array}\right]\; & =\left[\begin{array}{c} p\\ q\\ r \end{array}\right]\times \left[\begin{array}{c} pI_{Bxx}\\ qI_{Byy}\\ rI_{Bzz} \end{array}\right]\\ \; & =\left[\begin{array}{c} qr(I_{Bzz}-I_{Byy})\\ pr(I_{Bxx}-I_{Bzz})\\ pq(I_{Byy}-I_{Bxx}) \end{array}\right]. \end{array}$$

#### 4.2.4. Vehicle Angular Acceleration ${\dot{\omega }}_{B}$

Using the equations above, the vehicle’s angular acceleration ${\dot{\omega }}_{B}={\left[\dot{p}\;\dot{q}\;\dot{r}\right]}^{T}$ can be expressed as

(40) $$\begin{array}{cc} {\dot{\omega }}_{B} & =I_{B}^{-1}\left(\tau_{B}+\tau_{c}-\tau_{cori}+\tau_{ext}\right). \end{array}$$

Furthermore, neglecting external disturbance torques $\tau_{ext}$ acting on the vehicle and gyroscopic effects (i.e., $\tau_{ext}=0$), we have

(41) $$\begin{array}{cc} \left[\begin{array}{c} \dot{p}\\ \dot{q}\\ \dot{r} \end{array}\right] & =\left[\begin{array}{c} \frac{1}{I_{Bxx}}\left(\tau_{x}^{B}+\tau_{x}^{c}\right)+qr\left(\frac{I_{Byy}-I_{Bzz}}{I_{Bxx}}\right)\\ \frac{1}{I_{Byy}}\left(\tau_{y}^{B}+\tau_{y}^{c}\right)+pr\left(\frac{I_{Bzz}-I_{Bxx}}{I_{Byy}}\right)\\ \frac{1}{I_{Bzz}}\left(\tau_{z}^{B}+\tau_{z}^{c}\right)+pq\left(\frac{I_{Bxx}-I_{Byy}}{I_{Bzz}}\right) \end{array}\right]. \end{array}$$

### 4.3. Translational Motion

The thrust $T_{B}$ in the body coordinate system can be expressed using the thrust $T_{i}$ of each rotor as

(42) TB=∑i=12(RPiBTi).

Therefore, letting $T_{B}={\left[T_{x}\;T_{y}\;T_{z}\right]}^{T}$, we obtain

(43) $$\begin{array}{cc} T_{B}=\left[\begin{array}{c} T_{x}\\ T_{y}\\ T_{z} \end{array}\right] & =\left[\begin{array}{c} -C_{\alpha_{1}}S_{\beta_{1}}T_{1}-C_{\alpha_{2}}S_{\beta_{2}}T_{2}\\ S_{\alpha_{1}}T_{1}+S_{\alpha_{2}}T_{2}\\ -C_{\alpha_{1}}C_{\beta_{1}}T_{1}-C_{\alpha_{2}}C_{\beta_{2}}T_{2} \end{array}\right]\\ & =k_{f}\left[\begin{array}{c} -C_{\alpha_{1}}S_{\beta_{1}}\left({\bar{\omega }}_{1}^{2}+{\bar{\omega }}_{2}^{2}\right)-C_{\alpha_{2}}S_{\beta_{2}}\left({\bar{\omega }}_{3}^{2}+{\bar{\omega }}_{4}^{2}\right)\\ S_{\alpha_{1}}\left({\bar{\omega }}_{1}^{2}+{\bar{\omega }}_{2}^{2}\right)+S_{\alpha_{2}}\left({\bar{\omega }}_{3}^{2}+{\bar{\omega }}_{4}^{2}\right)\\ -C_{\alpha_{1}}C_{\beta_{1}}\left({\bar{\omega }}_{1}^{2}+{\bar{\omega }}_{2}^{2}\right)-C_{\alpha_{2}}C_{\beta_{2}}\left({\bar{\omega }}_{3}^{2}+{\bar{\omega }}_{4}^{2}\right) \end{array}\right]. \end{array}$$

Using the vehicle mass *m* and gravitational acceleration $g={\left[0\;0\;g\right]}^{T}$, the vehicle’s translational acceleration $\ddot{\xi }={\left[\ddot{x}\;\ddot{y}\;\ddot{z}\right]}^{T}$ can be expressed as

(44) ξ¨=g+1m(RBWTB+Fext),

(45) $$\begin{array}{cc} \left[\begin{array}{c} \ddot{x}\\ \ddot{y}\\ \ddot{z} \end{array}\right] & =\frac{1}{m}\left[\begin{array}{c} \begin{array}{c} C_{\theta }C_{\psi }T_{x}+\left(S_{\phi }S_{\theta }C_{\psi }-C_{\phi }S_{\psi }\right)T_{y}\\ \;+\left(C_{\phi }S_{\theta }C_{\psi }+S_{\phi }S_{\psi }\right)T_{z}\\ C_{\theta }S_{\psi }T_{x}+\left(S_{\phi }S_{\theta }S_{\psi }+C_{\phi }C_{\psi }\right)T_{y}\\ \;+\left(C_{\phi }S_{\theta }S_{\psi }-S_{\phi }C_{\psi }\right)T_{z}\\ -S_{\theta }T_{x}+S_{\phi }C_{\theta }T_{y}+C_{\phi }C_{\theta }T_{z}+mg \end{array} \end{array}\right] \end{array}$$

where $F_{ext}$ is the external disturbance force acting on the vehicle. Neglecting effects such as friction, we set $F_{ext}=0$.

## 5. Controller Design and Control Allocation

### 5.1. Computed Torque Method

The computed torque method is a technique to determine the generalized force (comprising thrust and torque in the body coordinate system) from the desired translational and angular accelerations in the world coordinate system, given that the vehicle’s physical information is known. Utilizing the relation $\dot{\eta }=W_{\eta }^{-1}\omega_{B}$ in the translational acceleration of Equation (44) and the rotational angular acceleration of Equation (40), and defining the generalized coordinates as $X≜{\left[\xi^{T}\eta^{T}\right]}^{T}$, the system can be represented as

(46) $$\begin{array}{cc} \ddot{X} & =F\left(\dot{X}\right)+G\left(X\right)u+D\left(X\right)d. \end{array}$$

This is because, from Equation (20),

(47) $$\begin{array}{cc} \ddot{\eta } & =\frac{d}{dt}\left(W_{\eta }^{-1}\right)\omega_{B}+W_{\eta }^{-1}{\dot{\omega }}_{B}\\ \; & =\frac{d}{dt}\left(W_{\eta }^{-1}\right)W_{\eta }\dot{\eta }+W_{\eta }^{-1}\left\{I_{B}^{-1}\left[\left(\tau_{B}+\tau_{c}\right)-\tau_{cori}+\tau_{ext}\right]\right\}\\ \; & =\frac{d}{dt}\left(W_{\eta }^{-1}\right)W_{\eta }\dot{\eta }-{\left(I_{B}W_{\eta }\right)}^{-1}\left[\left(W_{\eta }\dot{\eta }\right)\times I_{B}\left(W_{\eta }\dot{\eta }\right)\right]+{\left(I_{B}W_{\eta }\right)}^{-1}\left(\tau_{B}+\tau_{c}\right)+{\left(I_{B}W_{\eta }\right)}^{-1}\tau_{ext} \end{array}$$

and thus

$$\begin{array}{c} F\left(\dot{X}\right)=\left[\begin{array}{c} g\\ \frac{d}{dt}\left(W_{\eta }^{-1}\right)W_{\eta }\dot{\eta }-{\left(I_{B}W_{\eta }\right)}^{-1}\left[\left(W_{\eta }\dot{\eta }\right)\times I_{B}\left(W_{\eta }\dot{\eta }\right)\right] \end{array}\right], \end{array}$$

$$\begin{array}{c} G\left(X\right)=\left[\begin{array}{cc} {\frac{1}{m}}^{W}R_{B} & 0\\ 0 & {\left(I_{B}W_{\eta }\right)}^{-1} \end{array}\right],\;D\left(X\right)=\left[\begin{array}{cc} \frac{1}{m}I_{3\times 3} & 0\\ 0 & {\left(I_{B}W_{\eta }\right)}^{-1} \end{array}\right], \end{array}$$

$$\begin{array}{cc} u & ={\left[T_{B}^{T}\;{\left(\tau_{B}+\tau_{c}\right)}^{T}\right]}^{T},\;d={\left[F_{ext}^{T}\;\tau_{ext}^{T}\right]}^{T}. \end{array}$$

Here, since the rotation matrix RBW is orthogonal, RB−1W=WRBT. Also, as each principal moment of inertia $I_{B_{ii}}\neq 0\;\left(i=x,y,z\right)$, $G^{-1}\left(X\right)=\mathrm{diag}\left(m^{W}R_{B}^{T},W_{\eta }I_{B}\right)$. Then, the inverse system of Equation (46) is

(48) $$\begin{array}{cc} u & =G^{-1}\left(X\right)\left[\ddot{X}-F\left(\dot{X}\right)-D\left(X\right)d\right]. \end{array}$$

Let the target value for the generalized coordinate vector *X* be $X_{d}$. Constructing its augmented acceleration vector ${\ddot{X}}^{*}$ using a PD servo system yields

(49) $$\begin{array}{cc} {\ddot{X}}^{*} & ={\ddot{X}}_{d}+K_{d}\dot{e}+K_{p}e \end{array}$$

where $e=X_{d}-X$, $K_{d}>0$ is the derivative gain, and $K_{p}>0$ is the proportional gain. Substituting ${\ddot{X}}^{*}$ from Equation (49) for $\ddot{X}$ in Equation (48) gives

(50) $$\begin{array}{cc} u^{*} & =G^{-1}\left(X\right)\left[{\ddot{X}}_{d}+K_{d}\dot{e}+K_{p}e-F\left(\dot{X}\right)-D\left(X\right)d\right]. \end{array}$$

Substituting this $u^{*}$ into the original plant Equation (46) yields

(51) $$\begin{array}{c} \ddot{e}+K_{d}\dot{e}+K_{p}e=0. \end{array}$$

Here, we set $K_{p}=\mathrm{diag}\left(K_{p1},\ldots ,K_{p6}\right)$ and $K_{d}=\mathrm{diag}\left(K_{d1},\ldots ,K_{d6}\right)$. For application to actual vehicles, considering modeling errors, the condition $K_{di}=2\sqrt{K_{pi}}$, which ideally achieves a damping ratio of 1 (i.e., critical damping), is used as a base, but the damping condition for this error may need slight modification. In the simulations, the proportional gains for translational position and attitude angles are defined as $K_{p}=\mathrm{diag}(K_{px},K_{py},K_{pz},K_{p\phi },K_{p\theta },K_{p\psi })$, and the derivative gains as $K_{d}=\mathrm{diag}(K_{dx},K_{dy},K_{dz},K_{d\phi },K_{d\theta },K_{d\psi })$, with the following settings:

$$\begin{array}{cc} K_{px} & =K_{py}=3.0;\;K_{pz}=5;\;K_{p\phi }=K_{p\theta }=K_{p\psi }=2.0;\\ K_{dx} & =K_{dy}=3.46;\;K_{dz}=5;\;K_{d\phi }=K_{d\theta }=K_{d\psi }=2.83. \end{array}$$

### 5.2. Control Input Allocation Problem

Appropriate rotor speeds and tilt angles are allocated to each coaxial rotor to achieve the generalized force in the body coordinate system, determined earlier by the computed torque method. A single coaxial rotor constructed in this study utilizes two brushless motors. The thrust of coaxial rotor 1 is $T_{1}=k_{f}\left({\bar{\omega }}_{1}^{2}+{\bar{\omega }}_{2}^{2}\right)$, and that of coaxial rotor 2 is $T_{2}=k_{f}\left({\bar{\omega }}_{3}^{2}+{\bar{\omega }}_{4}^{2}\right)$. The reaction torque for the first rotor is $\tau_{c,1}=k_{t}\left({\bar{\omega }}_{2}^{2}-{\bar{\omega }}_{1}^{2}\right)$, and for the second rotor is $\tau_{c,2}=k_{t}\left({\bar{\omega }}_{3}^{2}-{\bar{\omega }}_{4}^{2}\right)$. However, directly solving for the rotation speeds ${\bar{\omega }}_{1}^{2}∼{\bar{\omega }}_{4}^{2}$ and tilt angles $\alpha_{i},\beta_{i},\left(i=1,2\right)$ results in an underdetermined problem: six generalized forces versus eight unknown decision variables. This would yield an effectiveness matrix of size 6×8, requiring solution via pseudo-inverse or numerical optimization such as constrained QP. Below, we present a simpler method based on experimental results for coaxial rotor thrust.

Referring to the experimental results on coaxial rotors by Itakura et al. [40], for a total thrust of $T_{total}=10.6$ N of the coaxial rotor, the thrust of the upstream propeller alone was $T_{upstream}=7.3$ N, and that of the downstream propeller alone was $T_{downstream}=8.0$ N. Therefore, the thrust efficiency is

(52) $$\begin{array}{cc} \eta_{thrust} & =\frac{T_{total}}{T_{upstream}+T_{downstream}}\\ \; & =\frac{10.6}{7.3+8.0}=0.693. \end{array}$$

This serves as an indicator of deviation from theoretical ideal conditions. On the other hand, the interference efficiency, which is the increase rate of total thrust relative to the upstream propeller’s thrust, is

(53) $$\begin{array}{cc} \eta_{if} & =\frac{T_{total}}{T_{upstream}}-1\\ \; & =\frac{10.6}{7.3}-1=0.452. \end{array}$$

Introducing this interference efficiency allows for the substitution of ${\bar{\omega }}_{2}^{2}$ with $\eta_{if}{\bar{\omega }}_{1}^{2}$ and ${\bar{\omega }}_{4}^{2}$ with $\eta_{if}{\bar{\omega }}_{3}^{2}$, thereby eliminating ${\bar{\omega }}_{2}^{2}$ and ${\bar{\omega }}_{4}^{2}$ from the unknown variables.

With this interference efficiency, the reaction torque $\tau_{c}$ from Equation (37) becomes

(54) $$\begin{array}{cc} \tau_{c} & =\left(\eta_{if}-1\right)k_{t}\left[\begin{array}{c} C_{\alpha_{1}}S_{\beta_{1}}{\bar{\omega }}_{1}^{2}-C_{\alpha_{2}}S_{\beta_{2}}{\bar{\omega }}_{3}^{2}\\ -S_{\alpha_{1}}{\bar{\omega }}_{1}^{2}+S_{\alpha_{2}}{\bar{\omega }}_{3}^{2}\\ C_{\alpha_{1}}C_{\beta_{1}}{\bar{\omega }}_{1}^{2}-C_{\alpha_{2}}C_{\beta_{2}}{\bar{\omega }}_{3}^{2} \end{array}\right]. \end{array}$$

Meanwhile, the rotational torque due to thrust $\tau_{B}$ from Equation (35) becomes

(55) $$\begin{array}{cc} \tau_{B} & =\left(\eta_{if}+1\right)k_{f}\left[\begin{array}{c} \begin{array}{c} \left(lC_{\alpha_{1}}C_{\beta_{1}}+h_{o}S_{\alpha_{1}}\right){\bar{\omega }}_{1}^{2}\\ \;-\left(lC_{\alpha_{2}}C_{\beta_{2}}-h_{o}S_{\alpha_{2}}\right){\bar{\omega }}_{3}^{2}\\ h_{o}C_{\alpha_{1}}S_{\beta_{1}}{\bar{\omega }}_{1}^{2}+h_{o}C_{\alpha_{2}}S_{\beta_{2}}{\bar{\omega }}_{3}^{2}\\ -lC_{\alpha_{1}}S_{\beta_{1}}{\bar{\omega }}_{1}^{2}+lC_{\alpha_{2}}S_{\beta_{2}}{\bar{\omega }}_{3}^{2} \end{array} \end{array}\right]. \end{array}$$

Similarly, the thrust $T_{B}$ from Equation (43) is rewritten as

(56) $$\begin{array}{c} T_{B}=\left(\eta_{if}+1\right)k_{f}\left[\begin{array}{c} -C_{\alpha_{1}}S_{\beta_{1}}{\bar{\omega }}_{1}^{2}-C_{\alpha_{2}}S_{\beta_{2}}{\bar{\omega }}_{3}^{2}\\ S_{\alpha_{1}}{\bar{\omega }}_{1}^{2}+S_{\alpha_{2}}{\bar{\omega }}_{3}^{2}\\ -C_{\alpha_{1}}C_{\beta_{1}}{\bar{\omega }}_{1}^{2}-C_{\alpha_{2}}C_{\beta_{2}}{\bar{\omega }}_{3}^{2} \end{array}\right]. \end{array}$$

Therefore, by introducing an intermediate 6-dimensional vector $n\;=\;{\left[n_{1a}\;n_{1b}\;n_{1c}\;n_{2a}\;n_{2b}\;n_{2c}\right]}^{T}$, defining $\Omega_{1}≜{\bar{\omega }}_{1}^{2}$, $\Omega_{2}≜{\bar{\omega }}_{3}^{2}$, and for i=1,2,

(57) $$\left\{\begin{array}{c} n_{ia}=\Omega_{i}C_{\alpha_{i}}S_{\beta_{i}}\\ n_{ib}=\Omega_{i}S_{\alpha_{i}}\\ n_{ic}=\Omega_{i}C_{\alpha_{i}}C_{\beta_{i}} \end{array}\right.,$$

the relation ${\left[T_{B}^{T}\;{\left(\tau_{B}+\tau_{c}\right)}^{T}\right]}^{T}$ finally reduces to a linear equation:

(58) $$\left[\begin{array}{c} T_{B}\\ \tau_{B}+\tau_{c} \end{array}\right]=An\left(\Omega_{i},\alpha_{i},\beta_{i}\right).$$

Here, the effectiveness matrix *A* is

(59) $$\begin{array}{c} A=\left[\begin{array}{cccccc} -\left(1+\eta_{if}\right)k_{f} & 0 & 0 & -\left(1+\eta_{if}\right)k_{f} & 0 & 0\\ 0 & \left(1+\eta_{if}\right)k_{f} & 0 & 0 & \left(1+\eta_{if}\right)k_{f} & 0\\ 0 & 0 & -\left(\eta_{if}+1\right)k_{f} & 0 & 0 & -\left(1+\eta_{if}\right)k_{f}\\ \left(\eta_{if}-1\right)k_{t} & \left(1+\eta_{if}\right)h_{o}k_{f} & \left(1+\eta_{if}\right)lk_{f} & -\left(1-\eta_{if}\right)k_{t} & \left(1+\eta_{if}\right)h_{o}k_{t} & -\left(1+\eta_{if}\right)lk_{f}\\ \left(1+\eta_{if}\right)h_{o}k_{f} & \left(1-\eta_{if}\right)k_{t} & 0 & \left(1+\eta_{if}\right)h_{o}k_{f} & \left(\eta_{if}-1\right)k_{t} & 0\\ -\left(1+\eta_{if}\right)lk_{f} & 0 & \left(\eta_{if}-1\right)k_{t} & \left(1+\eta_{if}\right)k_{f} & 0 & \left(1-\eta_{if}\right)k_{t} \end{array}\right]. \end{array}$$

**Figure 2:**
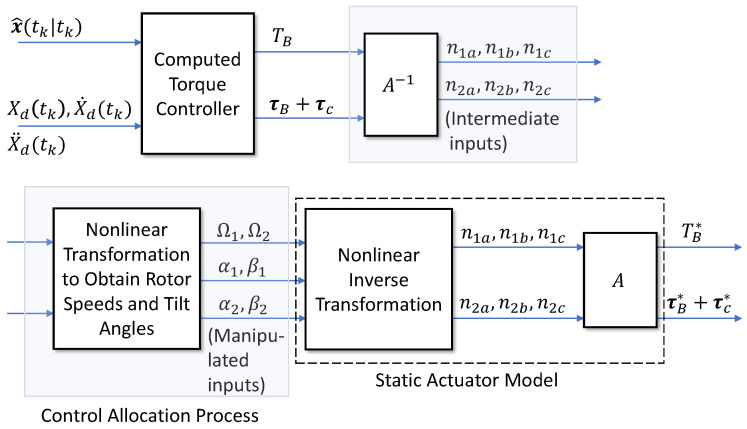
Control allocation process.

To generate control inputs for the actual vehicle, the intermediate variable vector n is obtained using $A^{-1}$, and then the squared rotation speeds $\Omega_{1}={\bar{\omega }}_{1}^{2}$, $\Omega_{2}={\bar{\omega }}_{3}^{2}$ and tilt angles $\left(\alpha_{1},\beta_{1}\right)$, $\left(\alpha_{2},\beta_{2}\right)$ for each rotor are generated via the following relations:

(60) $$\left\{\begin{array}{c} \Omega_{i}=\sqrt{n_{ia}^{2}+n_{ib}^{2}+n_{ic}^{2}}\\ \alpha_{i}=\arcsin\left(n_{ib}/\Omega_{i}\right)\\ \beta_{i}=\arctan\left(n_{ia}/n_{ic}\right) \end{array}\right..$$

Figure 2 illustrates the sequential calculation flow for this control allocation problem.

## 6. Preliminary Simulation: Application to the Falling Body Model

In this section, we describe state estimation for the falling body model [41] as a preliminary experiment. The falling body model is often used as a nonlinear model and was also employed as a simulation model by Takeno and Katayama [20]. The purpose here is to verify the basic effectiveness of the proposed method by performing state estimation under conditions similar to previous studies. Since the RK method used by Takeno et al. is a 4th-order discretization method, this section focuses on the 4th-order AB method for comparison.

### 6.1. Model Overview (Falling Body)

The differential equations for the falling body model are as follows [41]:

(61) $$\begin{array}{cc} {\dot{x}}_{1} & =x_{2}, \end{array}$$

(62) $$\begin{array}{cc} {\dot{x}}_{2} & =\frac{\rho_{0}e^{-x_{1}/k_{\rho }}x_{2}^{2}}{2\beta }-g. \end{array}$$

Here, $x_{1}$ is the altitude of the falling object, *g* is the gravitational acceleration, $\rho_{0}$ is the atmospheric density at sea level (0 m), $k_{\rho }$ is the decay coefficient, and β is the ballistic coefficient.

The extended state vector is defined as follows:

(63) $$\begin{array}{c} x=\left[\begin{array}{c} x_{1}\\ x_{2}\\ \beta \end{array}\right]=\left[\begin{array}{c} x_{1}\\ x_{2}\\ x_{3} \end{array}\right]\;\left(\mathrm{3-dimensional}\;\mathrm{state}\;\mathrm{vector}\right). \end{array}$$

Letting $f={\left[f_{1}\;f_{2}\;f_{3}\right]}^{T}$, the state–space model becomes

(64) $$\begin{array}{cc} \frac{dx_{1}}{dt} & ≜f_{1}=x_{2}, \end{array}$$

(65) $$\begin{array}{cc} \frac{dx_{2}}{dt} & ≜f_{2}=\frac{\rho_{0}}{2x_{3}}e^{-x_{1}/k_{\rho }}x_{2}^{2}-g, \end{array}$$

(66) $$\begin{array}{cc} \frac{dx_{3}}{dt} & ≜f_{3}=0. \end{array}$$

The observation equation is nonlinear and is assumed to be as follows:

(67) $$y=\sqrt{M_{1}^{2}+{\left(x_{1}-M_{2}\right)}^{2}}+v.$$

Here, $M_{1}$ is the horizontal distance between the falling object and the radar, and $M_{2}$ is the radar altitude. Furthermore, *v* is the observation noise, assumed to be Gaussian white noise with zero mean and variance *R*.

### 6.2. Estimation Algorithms (Falling Body)

In this study, we compare three types of numerical integration methods integrated with the UKF: the Euler method, the RK method, and the AB method. Hereafter, the UKF using the Euler method is called “Euler-UKF”, that using the RK method “RK-UKF”, and that using the AB method “AB-UKF”.

The detailed derivation and specific equations for the sigma point time update of each method are presented in Appendix C. This section provides only an overview of each method.

#### 6.2.1. Euler-UKF (Falling Body)

In Euler-UKF, the Euler method is applied to Equations (64)–(66). The Euler method has first-order accuracy and requires one function evaluation per update step. The specific sigma point time-update equations are given in Appendix C.1.

#### 6.2.2. RK-UKF (Falling Body)

In RK-UKF, the 4th-order RK method (hereafter denoted as RK4) is applied to Equations (64)–(66) [20]. The RK4 method has fourth-order accuracy and requires four function evaluations per update step. The specific sigma point time-update equations are given in Appendix C.2.

#### 6.2.3. AB-UKF (Falling Body)

In AB-UKF, the 4th-order AB method (hereafter denoted as AB4) is applied to Equations (64)–(66). The AB4 method achieves fourth-order accuracy while requiring only one function evaluation per update step. Instead, it stores and utilizes the function evaluation results for past sigma point matrices, $F_{c}\left(X_{k-1}\right)$, $F_{c}\left(X_{k-2}\right)$, and $F_{c}\left(X_{k-3}\right)$. The specific sigma point time-update equations are given in Appendix C.3.

### 6.3. Estimation Conditions (Falling Body)

State estimation simulations are performed using the three methods described above. The physical parameters are as follows: g=9.8 [m/s^2^], $\rho_{0}=2.202$ [kg·s^2^/m^4^], $k_{\rho }=1000/0.1558$ [m]. The true ballistic coefficient is β=2000 [kg/m^2^]. The horizontal distance between the falling object and the radar is $M_{1}$ = 10,000 [m], and the radar altitude is $M_{2}=0$ [m]. The step size is h=0.1 [s], the total number of sampling points is N=300, and the UT parameter is λ=2.0. The initial state vector, system noise covariance, and observation noise covariance are as follows [20]:

(68) $$\begin{array}{cc} \left[\begin{array}{c} x_{1}\left(0\right)\\ x_{2}\left(0\right)\\ x_{3}\left(0\right) \end{array}\right] & =\left[\begin{array}{c} 40000\;m\\ -3000\;m/s\\ 2000\;\mathrm{kg}/m^{2} \end{array}\right], \end{array}$$

(69) $$\begin{array}{cc} Q & =\mathrm{diag}(0,0,10\;{\mathrm{kg}}^{2}/m^{4}), \end{array}$$

(70) $$\begin{array}{cc} R & =3600\;m^{2}. \end{array}$$

The initial state estimate and its covariance matrix are as follows:

(71) $$\begin{array}{cc} \hat{x}\left(0|0\right) & =\left[\begin{array}{c} 42000\;m\\ -3100\;m/s\\ 3000\;\mathrm{kg}/m^{2} \end{array}\right], \end{array}$$

(72) $$\begin{array}{cc} P(0|0) & =\mathrm{diag}(10000\;m^{2},10000\;m^{2}/s^{2},10000\;{\mathrm{kg}}^{2}/m^{4}). \end{array}$$

### 6.4. Simulation Results

#### 6.4.1. State Estimation Accuracy

**Figure 3:**
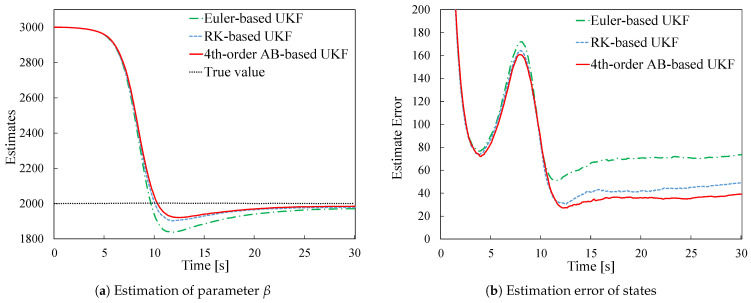
Comparison of estimation results.

Figure 3a shows the estimation results for the ballistic coefficient β, and Figure 3b shows the time evolution of the state estimation error. The state estimation error was calculated using the following RMSE (Root Mean Squared Error) formula:

(73) $$E\left(t_{k}\right)=\sqrt{\sum_{i=1}^{2}{\left(x_{i}\left(t_{k}\right)-{\hat{x}}_{i}\left(t_{k}|t_{k}\right)\right)}^{2}}.$$

The results are averages from 100 simulation runs for each method.

Figure 3a shows that the parameter β converges well for all three methods: Euler-UKF, RK-UKF, and AB-UKF. On the other hand, Figure 3b indicates that after the convergence of β, the state estimation error is suppressed more effectively in the order of Euler-UKF, RK-UKF, and AB-UKF.

Table 1 presents the average RMSE values of the state estimation error obtained from 100 simulation runs for each of the three methods. The computations were performed on a laptop with 16 GB RAM, an AMD Ryzen7 4800H with Radeon Graphics CPU, running Windows 10 and MATLAB 2022a.

As shown in Table 1, the Euler method exhibits the largest RMSE, while the RK and AB methods show smaller RMSE values than the Euler method. This result is attributed to the difference in discretization order: the Euler method has first-order accuracy, whereas the RK and AB methods have fourth-order accuracy.

#### 6.4.2. Computation Time

To more clearly demonstrate the differences between the methods, the estimation conditions from Section 6.3 were modified: h=0.01 and the total number of sampling points N=5000. Table 2 shows the comparison results for the total computation time of the entire UKF algorithm (from Equations (A3) to (A18)) and the computation time for the prediction step only (Equation (A9)).

Table 2 shows that the computation time is shortest for the Euler method, followed by the AB method, and then the RK method. The particularly short prediction time of the Euler method stems from the difference in discretization order.

### 6.5. Discussion

The results presented in Section 6.4 can be attributed to the following two factors.

1. **Small number of state variables**: The falling body model has three state variables, with $x_{3}$ representing a parameter estimation problem. Consequently, as is evident from the sigma point time-update equations, the discretization formulas are actually applied only to the state variables $x_{1}$ and $x_{2}$, making this a model where differences between the RK and AB methods are less likely to manifest.
2. **Small proportion of UT within the overall UKF algorithm**: The falling body model has a relatively small number of state variables and simple discretization equations, making it difficult for differences in computational load to become pronounced. As a result, the time spent on UT constitutes a small proportion of the overall UKF estimation algorithm, and the differences in computation time due to the discretization method are less noticeable.

Considering the above two points, the next section, Section 7, addresses a state estimation problem for a UAV model, which is higher dimensional than the falling body model.

## 7. Application to the Osprey-Type Drone

### 7.1. Model Overview (Drone)

The UAV vehicle conditions are based on the Osprey-type drone with 2-DOF tiltable coaxial rotors by Itakura et al. [40]. The vehicle’s general appearance and the derivation of its dynamic model have already been detailed in Section 4.

The vehicle’s position in the world coordinate system, $\xi ={\left[x\;y\;z\right]}^{T}$, follows the translational motion equation expressed by Equations (44) or (45).

On the other hand, the vehicle’s angular acceleration ${\dot{\omega }}_{B}={\left[\dot{p}\;\dot{q}\;\dot{r}\right]}^{T}$ is given by Equations (40) or (41). Assuming the Euler angle variations are relatively small, i.e., $W_{\eta }≃I$, then $p≃\dot{\phi },q≃\dot{\theta },r≃\dot{\psi }$. Therefore, Equation (41) can be rewritten as

(74) $$\left[\begin{array}{c} \ddot{\phi }\\ \ddot{\theta }\\ \ddot{\psi } \end{array}\right]=\left[\begin{array}{c} \frac{1}{I_{Bxx}}\left(\tau_{x}^{B}+\tau_{x}^{c}\right)+\dot{\theta }\dot{\psi }\left(\frac{I_{Byy}-I_{Bzz}}{I_{Bxx}}\right)\\ \frac{1}{I_{Byy}}\left(\tau_{y}^{B}+\tau_{y}^{c}\right)+\dot{\phi }\dot{\psi }\left(\frac{I_{Bzz}-I_{Bxx}}{I_{Byy}}\right)\\ \frac{1}{I_{Bzz}}\left(\tau_{z}^{B}+\tau_{z}^{c}\right)+\dot{\phi }\dot{\theta }\left(\frac{I_{Bxx}-I_{Byy}}{I_{Bzz}}\right) \end{array}\right].$$

Now, defining a 12-dimensional state vector as $x={\left[x\;\dot{x}\;y\;\dot{y}\;z\;\dot{z}\;\phi \;\dot{\phi }\;\theta \;\dot{\theta }\;\psi \;\dot{\psi }\right]}^{T}={\left[x_{1}\;x_{2}\;x_{3}\;x_{4}\;x_{5}\;x_{6}\;x_{7}\;x_{8}\;x_{9}\;x_{10}\;x_{11}\;x_{12}\right]}^{T}$, the continuous-time state equation for the UAV model can be derived from Equations (45) and (74) as follows:

(75) $$\begin{array}{c} \frac{d}{dt}\left[\begin{array}{c} x_{1}\\ x_{2}\\ x_{3}\\ x_{4}\\ x_{5}\\ x_{6}\\ x_{7}\\ x_{8}\\ x_{9}\\ x_{10}\\ x_{11}\\ x_{12} \end{array}\right]=\left[\begin{array}{c} x_{2}\\ A_{1}\left(x\right)T_{x}+A_{2}\left(x\right)T_{y}+A_{3}\left(x\right)T_{z}\\ x_{4}\\ A_{4}\left(x\right)T_{x}+A_{5}\left(x\right)T_{y}+A_{6}\left(x\right)T_{z}\\ x_{6}\\ A_{7}\left(x\right)T_{x}+A_{8}\left(x\right)T_{y}+A_{9}\left(x\right)T_{z}+g\\ x_{8}\\ \frac{1}{I_{Bxx}}\left(\tau_{x}^{B}+\tau_{x}^{c}\right)+x_{10}x_{12}\left(\frac{I_{Byy}-I_{Bzz}}{I_{Bxx}}\right)\\ x_{10}\\ \frac{1}{I_{Byy}}\left(\tau_{y}^{B}+\tau_{y}^{c}\right)+x_{8}x_{12}\left(\frac{I_{Bzz}-I_{Bxx}}{I_{Byy}}\right)\\ x_{12}\\ \frac{1}{I_{Bzz}}\left(\tau_{z}^{B}+\tau_{z}^{c}\right)+x_{8}x_{10}\left(\frac{I_{Bxx}-I_{Byy}}{I_{Bzz}}\right) \end{array}\right]. \end{array}$$

Here, $A_{1}\left(x\right),A_{2}\left(x\right),\ldots ,A_{9}\left(x\right)$ in Equation (75) are defined as follows:

(76) $$\left\{\begin{array}{c} A_{1}\left(x\right)=C_{x_{9}}C_{x_{11}}/m\\ A_{2}\left(x\right)=\left(S_{x_{7}}S_{x_{9}}C_{x_{11}}-C_{x_{7}}S_{x_{11}}\right)/m\\ A_{3}\left(x\right)=\left(C_{x_{7}}S_{x_{9}}C_{x_{11}}+S_{x_{7}}S_{x_{11}}\right)/m\\ A_{4}\left(x\right)=C_{x_{9}}S_{x_{11}}/m\\ A_{5}\left(x\right)=\left(S_{x_{7}}S_{x_{9}}S_{x_{11}}+C_{x_{7}}C_{x_{11}}\right)/m\\ A_{6}\left(x\right)=\left(C_{x_{7}}S_{x_{9}}C_{x_{11}}-S_{x_{7}}C_{x_{11}}\right)/m\\ A_{7}\left(x\right)=-S_{x_{9}}/m\\ A_{8}\left(x\right)=S_{x_{7}}C_{x_{9}}/m\\ A_{9}\left(x\right)=C_{x_{7}}C_{x_{9}}/m \end{array}\right..$$

### 7.2. Estimation Algorithms (Drone)

Similar to the falling body model, we compare three types of numerical integration methods integrated with the UKF for the UAV model: the Euler method, the RK method, and the AB method. The detailed derivation and specific equations for the sigma point time update of each method are presented in Appendix D. This section provides only an overview of each method.

#### 7.2.1. Euler-UKF (Drone)

In Euler-UKF, the Euler method is applied to Equation (75). The Euler method has first-order accuracy and requires one function evaluation per update step. The specific sigma point time-update equations are given in Appendix D.1.

#### 7.2.2. RK-UKF (Drone)

In RK-UKF, the RK4 method is applied to Equation (75). The RK4 method has fourth-order accuracy and requires four function evaluations per update step. The specific sigma point time-update equations are given in Appendix D.2.

#### 7.2.3. AB-UKF (Drone)

In AB-UKF, the AB method is applied to Equation (75). Regardless of its order, the AB method requires only one function evaluation per update step and stores and utilizes the function evaluation results for past sigma point matrices. The specific forms of the sigma point time-update equations corresponding to the 2nd to 6th order AB methods are given in Appendix D.3.

### 7.3. Estimation Conditions (Drone)

This section describes the UAV physical parameters and the covariance of system and observation noise used in the estimation simulation.

#### 7.3.1. Parameter Settings for the UAV Model

Similar to the falling body model, state estimation simulations are performed using the three methods described above. Table 3 summarizes the definitions and values of each parameter.

All initial states are set to $x\left(0\right)={\left[0\ldots 0\right]}^{T}$, and the initial estimation information is set to $\hat{x}\left(0|0\right)={\left[0\ldots 0\right]}^{T}$ and P(0|0)=diag(1,…,1).

#### 7.3.2. Determination of System Noise

In this simulation experiment, the system noise is set based on the global truncation error arising from discretization by the Euler, RK, and AB methods. When the step size is denoted by *h*, the global truncation error for the Euler method is proportional to $h^{1}$, and for the RK4 and AB4 methods, it is proportional to $h^{4}$. Therefore, letting the variance values of the respective system noises be $Q_{Euler}$ and $Q_{RK,AB4}$, they can be expressed as

(77) $$\begin{array}{cc} Q_{Euler} & =h\times I_{12\times 12}, \end{array}$$

(78) $$\begin{array}{cc} Q_{RK,AB4} & =h^{4}\times I_{12\times 12}. \end{array}$$

Thus, for comparison purposes in this simulation experiment, the value Q is set as follows:

(79) $$\begin{array}{c} Q=h^{5/2}\times I_{12\times 12}. \end{array}$$

Here, the exponent 5/2 signifies taking an intermediate order of magnitude between $Q_{Euler}$ and $Q_{RK,AB4}$.

#### 7.3.3. Determination of Observation Noise

Typically, UAVs are equipped with GPS sensors for position observation and gyro sensors for attitude observation. Determining a precise noise value for GPS sensor error is difficult due to various influencing factors; thus, for simplicity, the noise value is set to 2 m here. For the gyro sensor, we assume the use of the MPU-9250, a 9-axis sensor module from InvenSense.

The MPU-9250 datasheet states a gyro sensor noise value of 0.01 [deg/$\sqrt{\mathrm{Hz}}$]. Using this noise value and the step size *h* [s], the observation noise variance $R_{1}$ [rad^2^] for attitude angles ϕ,θ,ψ and $R_{2}$ [rad^2^/s^2^] for attitude angular velocities $\dot{\phi },\dot{\theta },\dot{\psi }$ are determined.

First, convert the dimension 0.01 [deg/$\sqrt{\mathrm{Hz}}$] to [rad/$\sqrt{\mathrm{Hz}}$] to obtain

(80) $$\begin{array}{c} 1.0\times 10^{-2}\left(\frac{\pi }{180}\right)\;\left[\mathrm{rad}/\sqrt{\mathrm{Hz}}\right]. \end{array}$$

Squaring the value obtained in Equation (80) yields

(81) $$\begin{array}{c} 1.0\times 10^{-4}{\left(\frac{\pi }{180}\right)}^{2}\;\left[{\mathrm{rad}}^{2}/\mathrm{Hz}\right]. \end{array}$$

Here, the dimension [Hz] can also be expressed as [1/s]; thus, the dimension [rad^2^/Hz] can also be expressed as [rad^2^·s]. Therefore, the observation noise variance $R_{1}$ [rad^2^] for attitude angles ϕ,θ,ψ is obtained by dividing Equation (81) by *h* [s]:

(82) $$\begin{array}{c} R_{1}=1.0\times 10^{-4}{\left(\frac{\pi }{180}\right)}^{2}/h\;\left[{\mathrm{rad}}^{2}\right]. \end{array}$$

Then, the observation noise variance $R_{2}$ [rad^2^/s^2^] for attitude angular velocities $\dot{\phi },\dot{\theta },\dot{\psi }$ is obtained by dividing Equation (82) by $h^{2}$ [s]:

(83) $$\begin{array}{c} R_{2}=1.0\times 10^{-4}{\left(\frac{\pi }{180}\right)}^{2}/h^{3}\;\left[{\mathrm{rad}}^{2}/s^{2}\right]. \end{array}$$

Consequently, the observation equation is

(84) $$\begin{array}{c} y\left(t_{k}\right)=Hx\left(t_{k}\right)+v\left(t_{k}\right), \end{array}$$

(85) $$\begin{array}{c} H=\left[\begin{array}{cc} H_{pos} & 0\\ 0 & I_{6\times 6} \end{array}\right] \end{array}$$

where $H_{pos}$ is the observation matrix for position (x,y,z):

(86) $$\begin{array}{c} H_{pos}=\left[\begin{array}{cccccc} 1 & 0 & 0 & 0 & 0 & 0\\ 0 & 0 & 1 & 0 & 0 & 0\\ 0 & 0 & 0 & 0 & 1 & 0 \end{array}\right]. \end{array}$$

The covariance R of the observation noise $v(t_{k})$ is represented by the following diagonal matrix:

(87) $$\begin{array}{c} R=\mathrm{diag}(2,\;2,\;2,\;R_{1},\;R_{2},\;R_{1},\;R_{2},\;R_{1},\;R_{2}). \end{array}$$

#### Remark

The observation setting used in this paper is linear (direct observation of position and attitude angles), and the advantages of nonlinear filtering are not fully utilized. In actual UAV navigation, nonlinear observations such as radar observations in GPS-denied environments are common. While the main focus of this paper is the analysis of the impact of state equation complexity on computational efficiency, and the linearity of observations is not an essential problem, extension to nonlinear observations is an important future task.

### 7.4. Target Trajectory

The simulation starts from the initial position $\left[x\;y\;z\right]={\left[0\;0\;0\right]}^{T}$ and initial attitude angles $\left[\phi \;\theta \;\psi \right]={\left[0\;0\;0\right]}^{T}$. First, over 10 s from the start time, the vehicle moves to the target position $\left[x_{d}\;y_{d}\;z_{d}\right]={\left[0\;0\;1\right]}^{T}$, while all target attitude angles remain zero. Subsequently, from 10 s to 70 s, the vehicle is commanded to follow the target position, velocity, and acceleration given below [42].

**Target position $X_{d}$**:

(88) $$\begin{array}{c} X_{d}=\left[\begin{array}{c} x_{d}\left(t\right)\\ y_{d}\left(t\right)\\ z_{d}\left(t\right)\\ \phi_{d}\left(t\right)\\ \theta_{d}\left(t\right)\\ \psi_{d}\left(t\right) \end{array}\right]=\left[\begin{array}{c} \sin\left(\frac{\pi }{10}\left(t-10\right)\right)\\ \cos\left(\frac{\pi }{4}\right)\left(-1+\cos\left(\frac{\pi }{10}\left(t-10\right)\right)\right)\\ -1+\sin\left(\frac{\pi }{4}\right)\left(-1+\cos\left(\frac{\pi }{10}\left(t-10\right)\right)\right)\\ \frac{\pi }{4}\sin\left(\frac{\pi }{40}\left(t-10\right)\right)\\ -\frac{\pi }{4}\sin\left(\frac{\pi }{40}\left(t-10\right)\right)\\ 0 \end{array}\right]. \end{array}$$

**Target velocity ${\dot{X}}_{d}$**:

(89) $$\begin{array}{c} {\dot{X}}_{d}=\left[\begin{array}{c} {\dot{x}}_{d}\left(t\right)\\ {\dot{y}}_{d}\left(t\right)\\ {\dot{z}}_{d}\left(t\right)\\ {\dot{\phi }}_{d}\left(t\right)\\ {\dot{\theta }}_{d}\left(t\right)\\ {\dot{\psi }}_{d}\left(t\right) \end{array}\right]=\left[\begin{array}{c} \frac{\pi }{10}\cos\left(\frac{\pi }{10}\left(t-10\right)\right)\\ -\frac{\pi }{10}\cos\left(\frac{\pi }{4}\right)\sin\left(\frac{\pi }{10}\left(t-10\right)\right)\\ -\frac{\pi }{10}\sin\left(\frac{\pi }{4}\right)\sin\left(\frac{\pi }{10}\left(t-10\right)\right)\\ \frac{\pi^{2}}{160}\cos\left(\frac{\pi }{40}\left(t-10\right)\right)\\ -\frac{\pi^{2}}{160}\cos\left(\frac{\pi }{40}\left(t-10\right)\right)\\ 0 \end{array}\right]. \end{array}$$

**Target acceleration ${\ddot{X}}_{d}$**:

(90) $$\begin{array}{c} {\ddot{X}}_{d}=\left[\begin{array}{c} {\ddot{x}}_{d}\left(t\right)\\ {\ddot{y}}_{d}\left(t\right)\\ {\ddot{z}}_{d}\left(t\right)\\ {\ddot{\phi }}_{d}\left(t\right)\\ {\ddot{\theta }}_{d}\left(t\right)\\ {\ddot{\psi }}_{d}\left(t\right) \end{array}\right]=\left[\begin{array}{c} -\frac{\pi^{2}}{100}\sin\left(\frac{\pi }{10}\left(t-10\right)\right)\\ -\frac{\pi^{2}}{100}\cos\left(\frac{\pi }{4}\right)\cos\left(\frac{\pi }{10}\left(t-10\right)\right)\\ -\frac{\pi^{2}}{100}\sin\left(\frac{\pi }{4}\right)\cos\left(\frac{\pi }{10}\left(t-10\right)\right)\\ -\frac{\pi^{3}}{6400}\sin\left(\frac{\pi }{40}\left(t-10\right)\right)\\ \frac{\pi^{3}}{6400}\sin\left(\frac{\pi }{40}\left(t-10\right)\right)\\ 0 \end{array}\right]. \end{array}$$

This target trajectory is a circular path with a radius of 1 m and center (x,y)=(0,−1) in the xy-plane, rotated 45 deg about the *x*-axis, and translated −1 m along the *z*-axis. Since this vehicle is left-right symmetric, independent control in three degrees of freedom (*x*, *y*, *z* directions) can be verified by following this circular path. The vehicle follows this trajectory with a period of 20 s per revolution. Simultaneously, the target roll angle changes every revolution: from 0 deg to 45 deg, 45 deg to 0 deg, and 0 deg to −45 deg. The target pitch angle changes every revolution: from 0 deg to −45 deg, −45 deg to 0 deg, and 0 deg to 45 deg. The target yaw angle is always 0 deg. Following this trajectory verifies the possibility of independent control in all six degrees of freedom.

### 7.5. Description of Comparative Experiments

This comparative experiment focuses on two aspects of the UKF based on each discretization method: “estimation accuracy” and “computational efficiency.” Details of the comparison methods are described below. Comparisons are performed in two patterns: “comparison of three methods: Euler, RK4, and AB4” and “comparison of 2nd to 6th-order AB methods.” The results of this comparative experiment are averages from 50 Monte Carlo simulations.

#### 7.5.1. Estimation Accuracy Comparison Experiment

The estimation accuracy comparison is performed by calculating the RMSE values between the estimates from each discretization-based UKF and the true values.

The RMSE value at estimation time *k* (k=1,…,N) is calculated using the following equations:

(91) $$\begin{array}{cc} {\mathrm{MSE}}_{x}\left(k\right) & =\frac{1}{k}\sum_{i=1}^{k}{\left(x\left(t_{i}\right)-\hat{x}\left(t_{i}|t_{i}\right)\right)}^{2}, \end{array}$$

(92) $$\begin{array}{cc} {\mathrm{RMSE}}_{x}\left(k\right) & =\sqrt{{\mathrm{MSE}}_{x}\left(k\right)} \end{array}$$

where $x(t_{i})$ and $\hat{x}\left(t_{i}|t_{i}\right)$ represent the true value and the estimate of position *x* at time *i*, respectively. Here, *N* is the total number of sampling points, determined from the simulation time of 70 s and the discretization period *h* [s] as N=70/h. For example, if h=0.01 [s], then N=7000. Equations (91) and (92) are similarly applied to other variables: positions y,z, velocities $\dot{x},\dot{y},\dot{z}$, attitude angles ϕ,θ,ψ, and attitude angular velocities $\dot{\phi },\dot{\theta },\dot{\psi }$.

Furthermore, when comparing estimation accuracy under different discretization periods (h=0.01,0.02,…,0.1), the sum of the individual RMSE values ${\mathrm{RMSE}}_{x}\left(N\right),{\mathrm{RMSE}}_{y}\left(N\right),$$\ldots ,{\mathrm{RMSE}}_{\dot{\psi }}\left(N\right)$ for the 12 state variables at the final discrete time k=N is denoted as RMSE(N), and this value is used for comparison.

Here, we discuss the theoretical meaning of RMSE(N). In the UAV model, when the a priori error covariance matrix $P(t_{k}|t_{k})$ at time $t_{k}$ is expressed as

(93) $$\begin{array}{c} P\left(t_{k}|t_{k}\right)=\left[\begin{array}{cccc} p_{1,1} & p_{1,2} & \ldots & p_{1,12}\\ p_{2,1} & p_{2,2} & \ldots & p_{2,12}\\ \vdots & \vdots & \ddots & \vdots \\ p_{12,1} & p_{12,2} & \ldots & p_{12,12} \end{array}\right], \end{array}$$

the trace of this error covariance matrix is denoted as $\mathrm{tr}(P\left(t_{k}|t_{k}\right))$. The aforementioned RMSE(N) is the sum of RMSE values based on actual sampled estimates, averaged over time up to k=N and averaged over the ensemble of 50 simulation results. Therefore, while transient values differ from the actual $\mathrm{tr}(P\left(t_{k}|t_{k}\right))$, they can be treated as equivalent values in the steady state.

#### 7.5.2. Computational Efficiency Comparison Experiment

The computational efficiency comparison experiment measures and compares computation times at the following three points:

- **Measurement Time I**: Computation time for sigma point time update (computation of Equation (A9)).
- **Measurement Time II**: Computation time for the entire UKF algorithm (computation from Equations (A3) to (A18)).
- **Measurement Time III**: Total computation time of the state estimation program for the UAV model.

**Figure 4:**
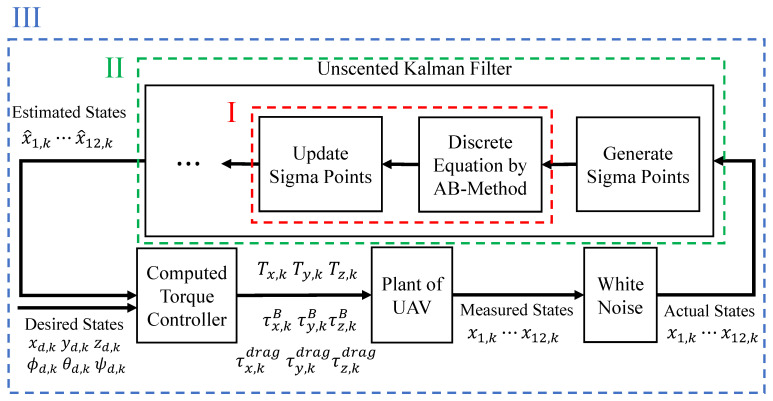
Overall diagram of the state estimation program for the UAV model and the location of measurement times I–III.

Figure 4 shows the overall diagram of the state estimation program for the UAV model and the locations of “Measurement Time I”, “Measurement Time II”, and “Measurement Time III”.

The controller design for generating generalized forces follows the computed torque method described in Section 5. The target trajectory involves a circular path from 10 s to 70 s, with yaw angle always at 0 deg, and roll and pitch angles changing to specified angles each revolution. However, the control inputs are first transformed from the generalized forces (thrust and torque for the motion system) provided by the computed torque method into actuator commands (rotor speeds and tilt angles) via the control allocation law. These are then multiplied by the propeller thrust coefficient $k_{f}$ and torque coefficient $k_{t}$ to reproduce the thrust and torque (including reaction torque) from the actuators, which are applied as inputs to the plant.

Below, we detail the data collection methods for Measurement Times I, II, and III.

- **Measurement Time I**: Since one simulation yields *N* measurement data points due to the total number of sampling points *N*, the average of all these is taken to calculate the average computation time per loop. However, due to the nature of the AB method, the average is taken over N−1 data points for 2nd order, N−2 for 3rd order, N−3 for 4th order, N−4 for 5th order, and N−5 for 6th order.
- **Measurement Time II**: Similar to Measurement Time I, the average computation time per loop is calculated, and the total computation time for *N* loops is also calculated.
- **Measurement Time III**: This is the total program computation time, so one simulation yields a single measurement data point.

Using these measurement methods, 50 simulations are repeatedly executed, and their average computation times are calculated for each case.

#### Remark

The purpose of this study’s computational efficiency evaluation is to compare algorithm-specific computational loads using the MATLAB environment. The validity of this approach is based on the following points:

- **Elimination of Hardware Dependence**: MATLAB simulations eliminate hardware-dependent factors such as CPU architecture, memory bandwidth, and cache size, enabling a pure evaluation of the relative computational load differences between the proposed numerical integration method (the AB method) and the conventional method (the RK method).
- **Indicator of Computational Amount**: In an onboard environment (flight control computer), the sampling frequency is high and computing resources are limited, making it important to reduce the number of floating-point operations and execution time to achieve the same accuracy. The reduction in computation time measured in MATLAB serves as a basic indicator of the effectiveness of processing speed improvement on an actual aircraft.

However, final verification requires implementation on an actual autopilot (e.g., Pixhawk) and benchmarking, which is considered a future challenge. This simulation demonstrates the theoretical and algorithmic advantages prior to actual implementation.

### 7.6. Results and Discussion of Estimation Accuracy

#### 7.6.1. Comparison of Three Methods in Estimation Accuracy: Euler, RK4, and AB4

**Figure 5:**
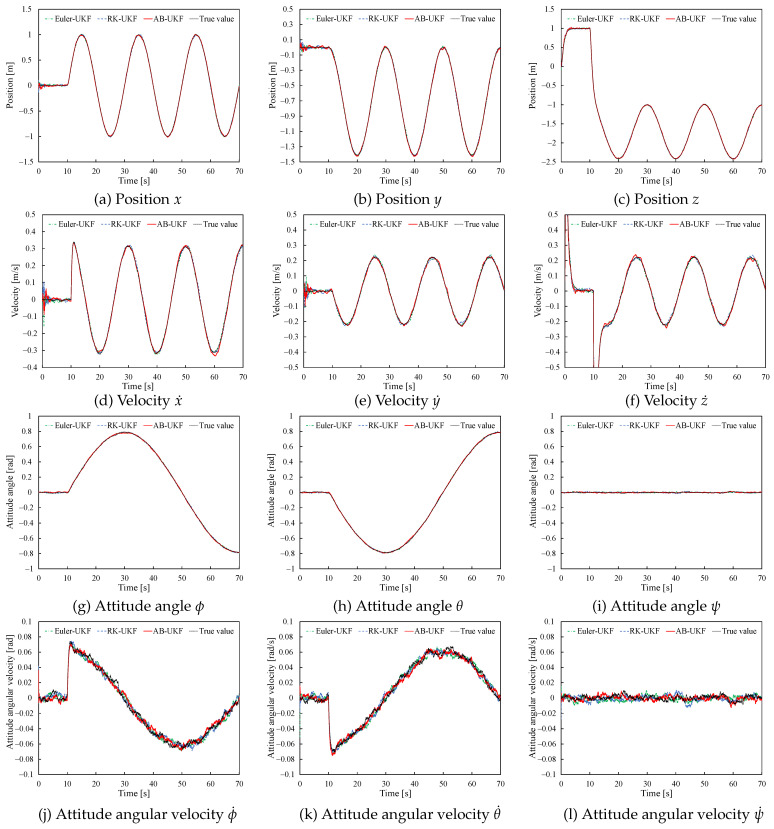
UKF estimates based on three discretization methods (h=0.01).

First, to show the estimates for the UAV model and to verify whether the UKF based on the multi-step AB method can perform estimation as stably as the single-step Euler and RK methods, Figure 5 shows the time evolution of state estimates by UKF based on the three methods for h=0.01.

As seen from Figure 5, the UKF estimates based on the AB method converge stably and achieve high-precision estimation. Also, no significant deviation from the RK method estimates is observed.

**Figure 6:**
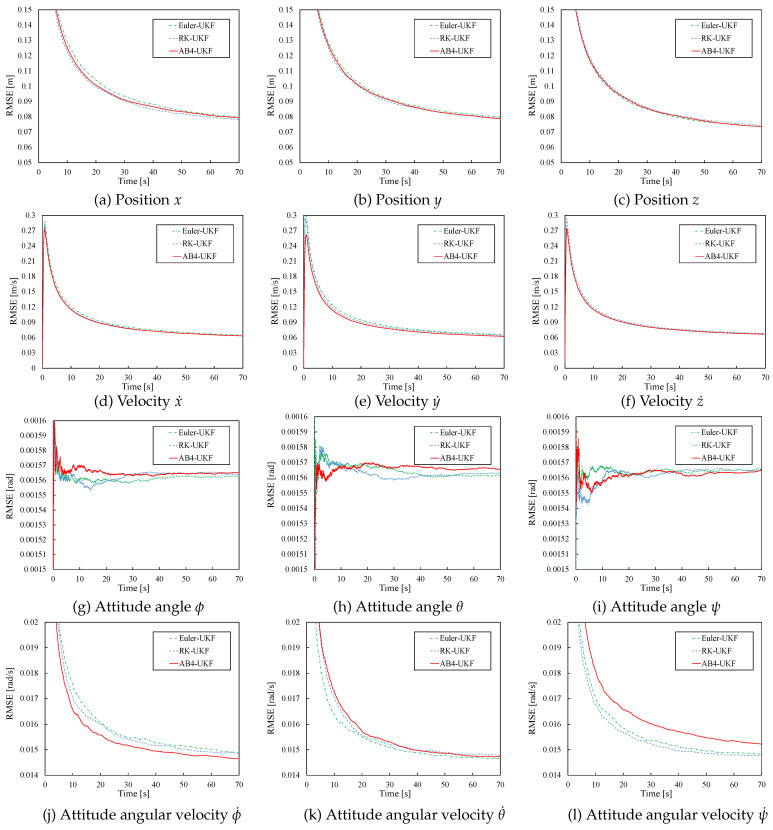
RMSE of UKF estimates based on three discretization methods (h=0.01).

Next, Figure 6 shows the time evolution of the RMSE values for the state estimates by UKF based on the three methods for h=0.01, over the 70 s simulation period. From Figure 6, under the condition h=0.01, although the RMSE differences among the methods are very small for all state variables, relatively larger differences are observed in the RMSE values for positions x,y, attitude angles ϕ,θ,ψ, and attitude angular velocities $\dot{\phi },\dot{\theta },\dot{\psi }$. Also, for all 12 state variables, the time variation of RMSE values becomes smaller after around 50 s of simulation time.

**Figure 7:**
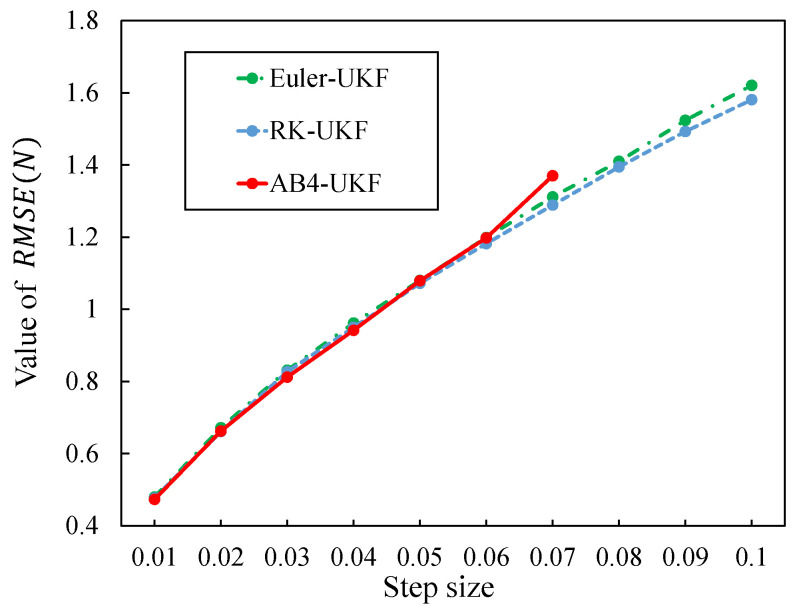
RMSE value change with step size change for three methods.

In addition, estimation accuracy is compared under different discretization periods: h=0.01,0.02,…,0.1. Figure 7 shows the change in RMSE(N) with varying discretization period *h*. Figure 7 shows that as the discretization period *h* increases, the differences among the methods become larger, and ultimately the RMSE(N) value for the RK method is the smallest. Furthermore, for the AB4 method, estimation values could not be obtained (diverged) for *h* values greater than or equal 0.08. Details regarding this are discussed later.

The specific values for each point in Figure 7 are shown in Table 4. The “N/A” in Table 4 indicates that estimation values could not be obtained due to divergence.

#### 7.6.2. RMSE Comparison of AB Methods with Different Orders of Accuracy

**Figure 8:**
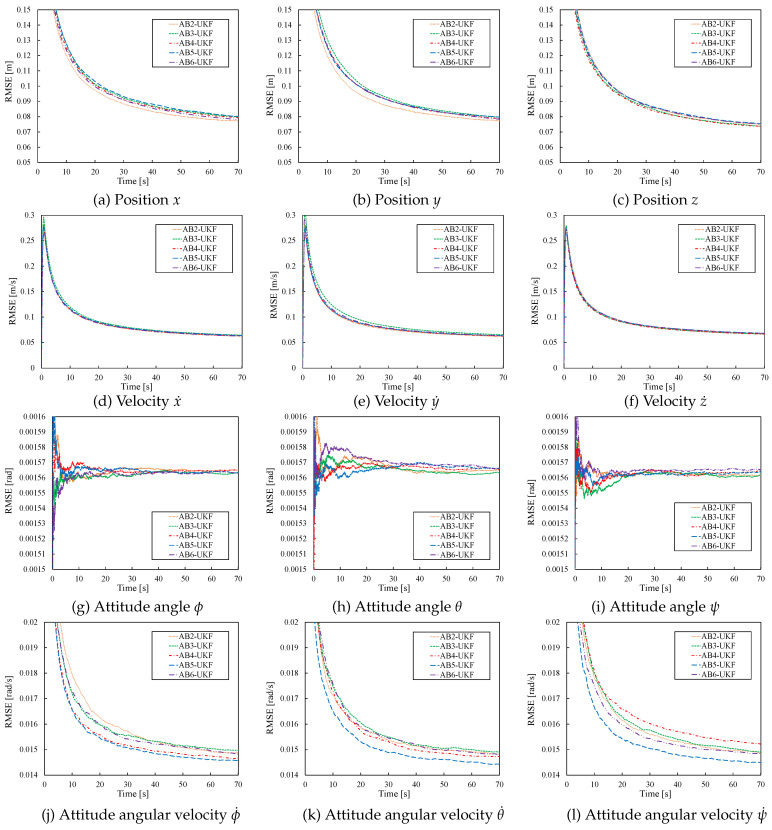
RMSE of UKF estimates for 2nd-order to 6th-order AB-based UKFs (h=0.01).

The estimation values are omitted, as their differences from Figure 6 were very small. Figure 8 shows the time evolution of the RMSE values for UKF estimates based on the second- to sixth-order AB methods for h=0.01. From Figure 8, under the condition h=0.01, all AB methods from the second to sixth order converge to values comparable to the RMSE values for UKF estimates based on the Euler and RK methods shown in Figure 6, without significant deviation.

**Figure 9:**
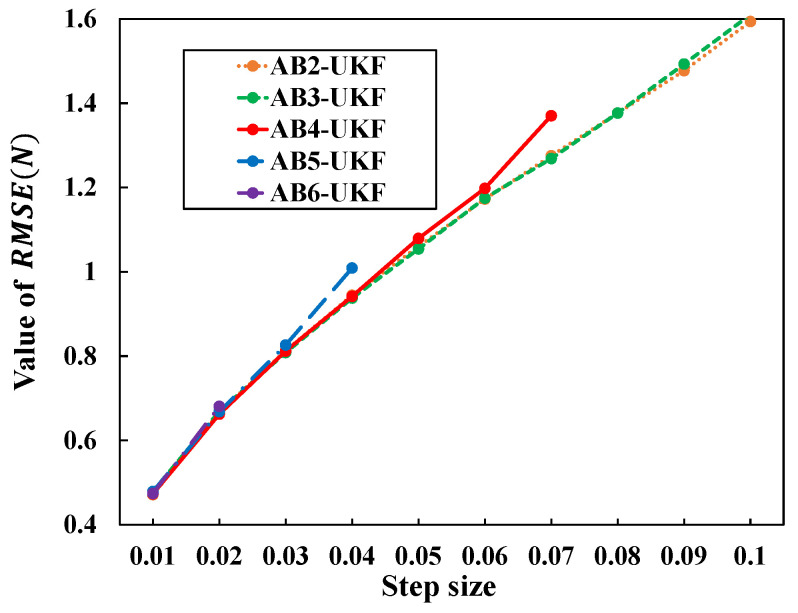
RMSE value change with step size change for different order AB-based UKFs.

Next, estimation accuracy is compared under different discretization periods: h=0.01,0.02,…,0.1. Figure 9 shows the change in RMSE(N) with varying discretization period *h*. Similar to Figure 7, Figure 9 shows that as the discretization period *h* increases, the differences among the methods become larger. Also, the sixth-order AB method diverged for *h* greater than or equal 0.03; the fifth-order, for *h* greater than or equal 0.05; and the fourth-order, for *h* greater than or equal 0.08, preventing estimation value acquisition.

The specific values for each point in Figure 9 are shown in Table 5. Additionally, for the 2nd and 3rd-order AB methods, further verification confirmed that they similarly diverge for values greater than or equal h=0.25 and h=0.15, respectively. Based on these results and additional experiments, the maximum step sizes at which each order can perform stable estimation without divergence are summarized as follows:

- **2nd-order AB method**: Stable up to h=0.24 (verified by additional experiments up to h=0.24). Divergence occurs at h≥0.25.
- **3rd-order AB method**: Stable up to h=0.14. Divergence occurs at h≥0.15.
- **4th-order AB method**: Stable up to h=0.07. Divergence occurs at h≥0.08.
- **5th-order AB method**: Stable up to h=0.04. Divergence occurs at h≥0.05.
- **6th-order AB method**: Stable up to h=0.02. Divergence occurs at h≥0.03.

These experimentally obtained stability limits are consistent with the theoretical property of linear multi-step methods that the absolute stability region shrinks as the order increases [36,37].

In practical applications, the choice of step size involves a trade-off between estimation accuracy and computational efficiency. A larger step size reduces computational load but may degrade accuracy or cause divergence, while a smaller step size improves stability and accuracy at the cost of increased computation time. Users should select the step size based on the specific requirements of their application, referring to the experimentally verified stability limits above as a guideline for the maximum allowable step size.

#### 7.6.3. Comparative Analysis: RK Method vs. AB Method

Comparing the results in Table 4 (re-entry vehicle problem) and Table 5 (UAV problem) reveals the following:

- **Accuracy Difference Between the RK and AB Methods**: At the same step size (e.g., h=0.01), the RMSE of the RK4-UKF (UAV: 0.47646) is nearly identical to the RMSE of the AB4-UKF (UAV: 0.47304), indicating no significant difference in accuracy between the two. This indicates that the RK method achieves high-order accuracy through multiple evaluations within a single step, while the AB method achieves similar accuracy with fewer function evaluations by utilizing past information.
- **Relationship between Order and Stability**: For both problems, the higher the order of the AB algorithm, the smaller the upper limit of the step size for stable estimation. This is due to the theoretical property of numerical analysis that the absolute stability region shrinks as the order of the linear multi-step algorithm increases.
- **Problem Complexity and Computational Efficiency**: For the simple 3D reentry vehicle problem (Table 2), the computational time of the AB4-based UKF increased by approximately 2.9% compared to the Euler-based UKF, while for the complex 12D UAV model (Table 6), it was reduced by approximately 5.1%. This reversal phenomenon is thought to be due to the characteristics of matrix operations in high-dimensional models (regularity of memory access patterns, cache efficiency).

These results indicate a trade-off between computational efficiency and numerical stability. When applying this method to real problems, it is necessary to select an appropriate order based on the required estimation accuracy and acceptable step size.

### 7.7. Results and Discussion of Computational Efficiency

#### 7.7.1. Comparison of Three Methods in Computational Efficiency: Euler, RK4, and AB4

Table 6 shows the computation times for Time I, Time II, and Time III for the three methods (Euler, RK4, and AB4) with step size h=0.01. From Table 6, the reduction rate in computation time for the 4th-order AB method is 12.3% compared to Euler and 41.1% compared to RK for Time I; 7.1% compared to Euler and 29.9% compared to RK4 for Time II; and 5.1% compared to Euler and 20.9% compared to RK4 for Time III.

**Figure 10:**
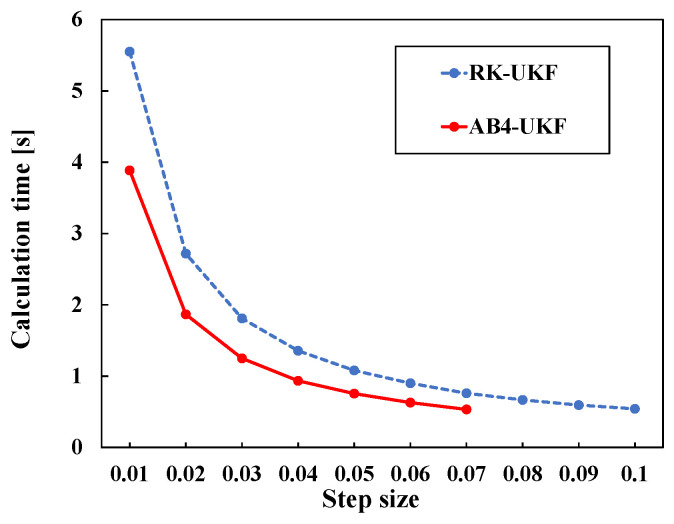
Calculation time change with step size change for RK4-based UKF and AB4-based UKF.

Next, Figure 10 shows the change in computation time with varying discretization period *h*. Figure 10 shows that as *h* becomes smaller, the difference between the two methods increases. This is considered to be due to the influence of the increasing number of program loops as the total number of sampling points *N* becomes larger for smaller *h*.

#### 7.7.2. Computational Time Comparison of AB Methods with Different Orders of Accuracy

Next, computation times for Time I, Time II, and Time III are compared under the same conditions for AB methods from 2nd to 6th order for h=0.01, not just the AB4 method. The result is given in Table 7. The computational difference among AB methods from 2nd to 6th order lies only in the number of past sigma point matrices used as “previous information”. Therefore, the differences among methods are smaller than the results shown in Table 6.

**Figure 11:**
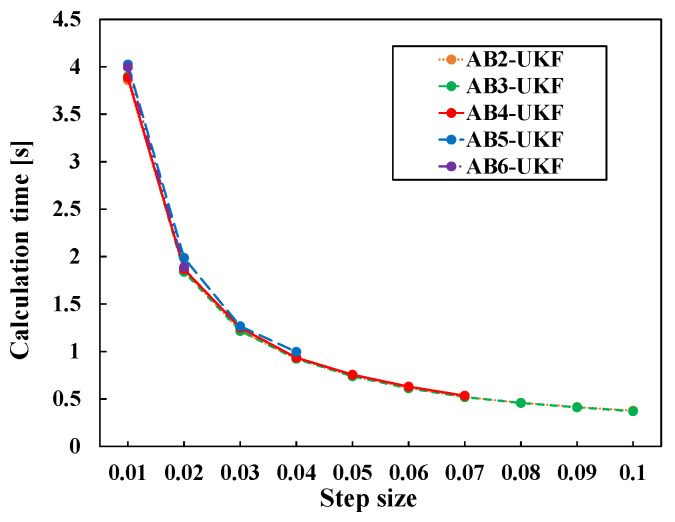
Calculation time change with step size change for different order AB-based UKFs.

Next, Figure 11 shows the change in computation time with varying discretization period *h*. From Figure 11, unlike the comparison between RK and AB methods, almost no difference is seen among the methods. The reason for this result is considered to be that the difference in computational load due to the order of the AB method stems only from the difference in the number of sigma point matrices handled, and the number of derivative calculations per step is the same for any order. Also, from this result, it can be understood that while estimation with the AB method diverges if the step size is too large, it does not diverge under conditions with *h* values smaller than 0.01, allowing estimation with higher-order accuracy and shorter computation time compared to the RK method under those conditions.

## 8. Conclusions

This paper proposed a UKF that newly integrates the Adams–Bashforth method into the sigma point time-update equation of the UKF (AB-UKF) as an alternative to the Runge–Kutta method. The proposed method maintains estimation accuracy comparable to the RK-UKF proposed by Takeno and Katayama [20] while enabling estimation with more efficient computation time.

The main findings obtained from the numerical simulation results are summarized below.

- **Maintenance of estimation accuracy**: For both models, AB-UKF achieved estimation accuracy comparable to RK-UKF. For the UAV model (h=0.01), no significant difference was observed between the RMSE of AB4-UKF (0.47304) and that of RK4-UKF (0.47646).
- **Comparison of computational efficiency**: Comparison of total computation time relative to the Euler-based UKF confirmed the effectiveness of the proposed method.
  – For the simple three-dimensional reentry vehicle problem (Table 2), the computation time of AB4-based UKF was approximately 2.9% longer than Euler-based UKF (slower than Euler), whereas RK4-based UKF was approximately 9.4% longer.
  – For the complex 12-dimensional UAV model (Table 6), the computation time of AB4-based UKF was approximately 5.1% shorter than Euler-based UKF (faster than Euler). In contrast, RK4-based UKF exhibited a more pronounced slowdown, with an approximately 20.0% increase.
  These results demonstrate that *the computational time reduction effect achieved by the proposed AB method integration becomes more pronounced for models with more complex state equations and higher dimensions*.
- **Relationship between order and stability**: Verification using the UAV model quantitatively showed that higher-order AB methods have a smaller upper limit on the step size that allows stable estimation. The maximum stable step sizes for each order are: AB2: h≤0.24, AB3: h≤0.14, AB4: h≤0.07, AB5: h≤0.04, and AB6: h≤0.02. Divergence occurs at step sizes exceeding these limits.

These results indicate that high-precision numerical integration methods are not necessarily superior in terms of computational efficiency; the RK4-based UKF was consistently slower than the Euler-based UKF in both problems (reentry problem: +9.4%, UAV problem: +20.0%). The proposed AB-UKF is an effective option for improving computational efficiency while maintaining estimation accuracy, particularly for high-dimensional models.

### 8.1. Limitations of This Study

This study has the following limitations:

- **Linearity of observations**: The observations in the UAV model are linear (direct observation of position and attitude angles), and the advantages of nonlinear filtering are not fully utilized. In actual UAV navigation, nonlinear observations such as radar observations in GPS-denied environments are common.
- **Simulation environment**: The evaluation of computational efficiency is based on MATLAB simulations and has not been verified for real-time operation on actual hardware (flight control computers).
- **Application to specific models**: The effectiveness of this method has been verified on two models (the falling body model and the UAV model), but additional verification is required for generalization to other nonlinear systems.

### 8.2. Future Work

Based on the above limitations, future work includes the following:

- **Extension to nonlinear observations**: Verify the effectiveness of the proposed method under nonlinear observations using only range and azimuth. Specifically, we will tackle the problem of estimating a 12-dimensional state vector using only range and azimuth information from one or two radars, and verify whether AB-UKF can maintain its advantage in terms of computational efficiency even under nonlinear observations.
- **Reduction of the number of sigma points**: While the standard UKF uses (2n+1) sigma points, the number can be reduced to n+2 or n+1 by using the Spherical Simplex Unscented Transformation [15,43,44,45] or the Simplex Unscented Transformation [46]. Combining these methods with the AB method is expected to further improve computational efficiency.
- **Implementation on actual hardware**: Implement the proposed algorithm on a microcontroller (e.g., Pixhawk) for the UAV model used in this simulation, and verify the degree of improvement in estimation accuracy and computational efficiency in actual flight environments.
- **Adaptive step size control**: Integrate the adaptive step size control based on the degree of nonlinearity proposed by Wang et al. [25] into AB-UKF to further optimize estimation accuracy and computational efficiency.

The proposed method provides a new option for the trade-off between computational efficiency and estimation accuracy in state estimation for high-dimensional nonlinear systems, and its future development is expected to lead to applications in practical UAV navigation systems.

## Figures and Tables

**Table 1:** Comparison of RMSE among the three methods.

	Method		
	Euler-UKF	RK-UKF	AB4-UKF
RMSE	125.585	116.826	115.537

**Table 2:** Comparison of computation time among the three methods.

	Method		
	Euler-UKF	RK-UKF	AB4-UKF
Alg. total time ($\times 10^{-2}$) [s]	8.4612	9.2542	8.7108
Pred. time only ($\times 10^{-6}$) [s]	58.3842	77.8698	70.6750

**Table 3:** Simulation parameters of the UAV.

Variable	Definition	Value
*m* [kg]	Mass of UAV	1.5
*g* [m/s^2^]	Acceleration of gravity	9.81
$I_{Bxx}$ [kg·m^2^]	Moment of inertia around $X_{B}$ axis	0.01
$I_{Byy}$ [kg·m^2^]	Moment of inertia around $Y_{B}$ axis	0.01
$I_{Bzz}$ [kg·m^2^]	Moment of inertia around $Z_{B}$ axis	0.006
*l* [m]	$Y_{B}$ axis distance between $O_{B}$ and $O_{P_{i}}$	0.24
$h_{o}$ [m]	$Z_{B}$ axis distance between $O_{B}$ and $O_{P_{i}}$	0.045
$k_{f}$ [Ns^2^/rad^2^]	Thrust coefficient of the propeller	$1.784\times 10^{-5}$
$k_{t}$ [Nms^2^/rad^2^]	Drag coefficient of the propeller	$4.379\times 10^{-7}$

**Table 4:** Comparison of RMSE(N) with different step size for three methods.

	Method		
	Euler	RK4	AB4
h=0.01	0.47961	0.47646	0.47304
h=0.02	0.67167	0.66121	0.66235
h=0.03	0.83102	0.82695	0.81186
h=0.04	0.96179	0.94971	0.94171
h=0.05	1.07992	1.07212	1.07913
h=0.06	1.19891	1.18197	1.19785
h=0.07	1.31153	1.28889	1.37018
h=0.08	1.41016	1.39465	N/A
h=0.09	1.52378	1.49280	N/A
h=0.10	1.62067	1.58091	N/A

N/A indicates that the computation could not be performed.

**Table 5:** RMSE(N) values for 2nd- to 6th-order AB-based UKFs with different step sizes.

	AB Method with Different Order				
	AB2	AB3	AB4	AB5	AB6
h=0.01	0.47140	0.47891	0.47304	0.47878	0.47409
h=0.02	0.66619	0.66920	0.66235	0.66816	0.68082
h=0.03	0.81327	0.80840	0.81186	0.82628	N/A
h=0.04	0.94432	0.93755	0.94171	1.00880	N/A
h=0.05	1.06020	1.05421	1.07913	N/A	N/A
h=0.06	1.17243	1.17460	1.19785	N/A	N/A
h=0.07	1.27515	1.26857	1.37018	N/A	N/A
h=0.08	1.37661	1.37650	N/A	N/A	N/A
h=0.09	1.47709	1.49280	N/A	N/A	N/A
h=0.10	1.59363	1.60998	N/A	N/A	N/A

N/A: Not available (calculation could not be performed).

**Table 6:** Comparison of computational time among Euler-based UKF, RK4-based UKF and AB4-based UKF (h=0.01).

	Method		
	Euler	RK4	AB4
Time I ($\times 10^{-4}$) [s]	3.7593	5.6078	3.2978
Time II ($\times 10^{-4}$) [s]	6.0411	8.0064	5.6144
Time III [s]	6.3785	7.6520	6.0550

**Table 7:** Comparison of computational time for 2nd-order to 6th-order AB-based UKFs.

	AB Method with Different Order				
	AB2	AB3	AB4	AB5	AB6
Time I ($\times 10^{-4}$) [s]	3.2125	3.2839	3.3212	3.3478	3.4263
Time II ($\times 10^{-4}$) [s]	5.5107	5.6166	5.6353	5.6081	5.7562
Time III [s]	6.1799	6.2990	6.1856	6.1427	6.3341

## Data Availability

The data presented in this study are available on request from the corresponding author.

## Appendix A. Unscented Kalman Filter Algorithm

This appendix presents the detailed computational procedure of the UKF algorithm outlined in Section 2.

### Appendix A.1. Problem Formulation

Consider the following discrete-time state–space model:

(A1) $$\begin{array}{cc} x_{k+1} & =f\left(x_{k},u_{k}\right)+w_{k}, \end{array}$$

(A2) $$\begin{array}{cc} y_{k} & =h\left(x_{k}\right)+v_{k} \end{array}$$

where $x_{k}\in R^{n}$ is the state vector, $u_{k}\in R^{m}$ is the input vector, and $y_{k}\in R^{p}$ is the output vector. $w_{k}\in R^{n}$ and $v_{k}\in R^{p}$ are Gaussian white noises with zero mean and covariance matrices *Q* and *R*, respectively.

### Appendix A.2. UKF Algorithm

Given the initial estimate ${\hat{x}}_{0|0}$ and initial covariance matrix $P_{0|0}$, the following procedure is executed at each time step k=0,1,….

#### Appendix A.2.1. Prediction Step

**Step 1: Generation of sigma points**

(A3) $$\begin{array}{cc} X_{0,k|k} & ={\hat{x}}_{k|k}, \end{array}$$

(A4) $$\begin{array}{cc} X_{i,k|k} & ={\hat{x}}_{k|k}+{\left(\sqrt{\left(n+\lambda \right)P_{k|k}}\right)}_{i},\;i=1,\ldots ,n, \end{array}$$

(A5) $$\begin{array}{cc} X_{i+n,k|k} & ={\hat{x}}_{k|k}-{\left(\sqrt{\left(n+\lambda \right)P_{k|k}}\right)}_{i},\;i=1,\ldots ,n. \end{array}$$

Here, ${\left(\sqrt{\left(n+\lambda \right)P_{k|k}}\right)}_{i}$ denotes the *i*-th column of the matrix square root of $\left(n+\lambda \right)P_{k|k}$. $\lambda =\alpha^{2}\left(n+\kappa \right)-n$ is a scaling parameter, where α determines the spread of the sigma points (typically $10^{-4}\leq \alpha \leq 1$), and κ is a secondary scaling parameter (typically 0 or 3−n).

The corresponding weights are as follows:

(A6) $$\begin{array}{cc} W_{0}^{\left(m\right)} & =\frac{\lambda }{n+\lambda }, \end{array}$$

(A7) $$\begin{array}{cc} W_{0}^{\left(c\right)} & =\frac{\lambda }{n+\lambda }+\left(1-\alpha^{2}+\beta \right), \end{array}$$

(A8) $$\begin{array}{cc} W_{i}^{\left(m\right)}=W_{i}^{\left(c\right)} & =\frac{1}{2(n+\lambda )},\;i=1,\ldots ,2n \end{array}$$

where β is a parameter used to incorporate prior knowledge of the distribution; for Gaussian distributions, β=2 is optimal.

**Step 2: Time update of sigma points**

(A9)
$$\begin{array}{cc} X_{i,k+1|k} & =f(X_{i,k|k},u_{k}),\;i=0,\ldots ,2n. \end{array}$$

**Step 3: Computation of predicted state and predicted covariance**

(A10)
$$\begin{array}{cc} {\hat{x}}_{k+1|k} & =\sum_{i=0}^{2n}W_{i}^{\left(m\right)}X_{i,k+1|k}, \end{array}$$

(A11)
$$\begin{array}{cc} P_{k+1|k} & =\sum_{i=0}^{2n}W_{i}^{\left(c\right)}\left(X_{i,k+1|k}-{\hat{x}}_{k+1|k}\right){\left(X_{i,k+1|k}-{\hat{x}}_{k+1|k}\right)}^{T}+Q. \end{array}$$

#### Appendix A.2.2. Update Step

**Step 4: Generation of output sigma points**

(A12)
$$\begin{array}{cc} Y_{i,k+1|k} & =h(X_{i,k+1|k}),\;i=0,\ldots ,2n. \end{array}$$

**Step 5: Computation of predicted output**

(A13)
$$\begin{array}{cc} {\hat{y}}_{k+1|k} & =\sum_{i=0}^{2n}W_{i}^{\left(m\right)}Y_{i,k+1|k}. \end{array}$$

**Step 6: Computation of output covariance and cross-covariance**

(A14)
$$\begin{array}{cc} P_{yy} & =\sum_{i=0}^{2n}W_{i}^{\left(c\right)}\left(Y_{i,k+1|k}-{\hat{y}}_{k+1|k}\right){\left(Y_{i,k+1|k}-{\hat{y}}_{k+1|k}\right)}^{T}+R, \end{array}$$

(A15)
$$\begin{array}{cc} P_{xy} & =\sum_{i=0}^{2n}W_{i}^{\left(c\right)}\left(X_{i,k+1|k}-{\hat{x}}_{k+1|k}\right){\left(Y_{i,k+1|k}-{\hat{y}}_{k+1|k}\right)}^{T}. \end{array}$$

**Step 7: Computation of Kalman gain**

(A16)
$$\begin{array}{cc} K_{k+1} & =P_{xy}P_{yy}^{-1}. \end{array}$$

**Step 8: Update of state estimate and covariance matrix**

(A17)
$$\begin{array}{cc} {\hat{x}}_{k+1|k+1} & ={\hat{x}}_{k+1|k}+K_{k+1}\left(y_{k+1}-{\hat{y}}_{k+1|k}\right), \end{array}$$

(A18)
$$\begin{array}{cc} P_{k+1|k+1} & =P_{k+1|k}-K_{k+1}P_{yy}K_{k+1}^{T}. \end{array}$$

The above constitutes the standard algorithm for the DD-UKF. In this study, we apply the Adams–Bashforth method described in Section 3 to the time update *f* in Equation (A9).

## Appendix B. Details of the Runge–Kutta Method

This appendix provides the detailed derivation and specific equations for the integration of the Runge–Kutta method with the UT described in Section 3.2.

### Appendix B.1. General Form of the 4th-Order Runge–Kutta Method

The numerical integration of the ordinary differential equation $\dot{x}=f_{c}\left(x\right)$ using the 4th-order Runge–Kutta method is given by the following formulas:

(A19) $$\begin{array}{cc} k_{1} & =f_{c}\left(x_{k}\right), \end{array}$$

(A20) $$\begin{array}{cc} k_{2} & =f_{c}\left(x_{k}+\frac{h}{2}k_{1}\right), \end{array}$$

(A21) $$\begin{array}{cc} k_{3} & =f_{c}\left(x_{k}+\frac{h}{2}k_{2}\right), \end{array}$$

(A22) $$\begin{array}{cc} k_{4} & =f_{c}\left(x_{k}+hk_{3}\right), \end{array}$$

(A23) $$\begin{array}{cc} x_{k+1} & =x_{k}+\frac{h}{6}\left(k_{1}+2k_{2}+2k_{3}+k_{4}\right). \end{array}$$

### Appendix B.2. Extension to the Sigma Point Matrix

When applying the 4th-order RK method to the UKF sigma point matrix $X_{k}$ (Equation (7)), the following coefficients are defined for each sigma point *j* and each state variable *i*:

(A24) $$\begin{array}{cc} a_{1,ij} & =f_{i}\left(X_{1,j,k},\ldots ,X_{n,j,k},t_{k}\right), \end{array}$$

(A25) $$\begin{array}{cc} a_{2,ij} & =f_{i}\left(X_{1,j,k}+\frac{h}{2}a_{1,1j},\ldots ,X_{n,j,k}+\frac{h}{2}a_{1,nj},t_{k}+\frac{h}{2}\right), \end{array}$$

(A26) $$\begin{array}{cc} a_{3,ij} & =f_{i}\left(X_{1,j,k}+\frac{h}{2}a_{2,1j},\ldots ,X_{n,j,k}+\frac{h}{2}a_{2,nj},t_{k}+\frac{h}{2}\right), \end{array}$$

(A27) $$\begin{array}{cc} a_{4,ij} & =f_{i}\left(X_{1,j,k}+ha_{3,1j},\ldots ,X_{n,j,k}+ha_{3,nj},t_{k}+h\right). \end{array}$$

Using these coefficients, the time-update equation for the sigma points is expressed as follows:

(A28) $$\begin{array}{c} X_{i,j,k+1}^{-}=X_{i,j,k}+\frac{h}{6}\left(a_{1,ij}+2a_{2,ij}+2a_{3,ij}+a_{4,ij}\right) \end{array}$$

where i=1,…,n and j=1,…,2n+1. Equation (A28) corresponds to Equation (8) in the main text.

## Appendix C. Details of Time-Update Equations for the Falling Body Model

This appendix presents the specific sigma point time-update equations for each method outlined in Section 6.2.

### Appendix C.1. Time-Update Equations for Euler-UKF (Falling Body)

Applying the Euler method to Equations (64)–(66) yields the following sigma point time-update equations:

(A29) $$\begin{array}{cc} X_{1,j,k+1}^{-} & =X_{1,j,k}+hX_{2,j,k}, \end{array}$$

(A30) $$\begin{array}{cc} X_{2,j,k+1}^{-} & =X_{2,j,k}+h\left(\frac{\rho_{0}}{2X_{3,j,k}}e^{-X_{1,j,k}/k_{\rho }}X_{2,j,k}^{2}-g\right), \end{array}$$

(A31) $$\begin{array}{cc} X_{3,j,k+1}^{-} & =X_{3,j,k}, \end{array}$$

where j=1,…,7.

### Appendix C.2. Time-Update Equations for RK-UKF (Falling Body)

To apply the 4th-order Runge–Kutta method to Equations (64)–(66), the following coefficients are defined [20]:

(A32) $$\begin{array}{cc} c_{1} & =X_{2,j,k}, \end{array}$$

(A33) $$\begin{array}{cc} q_{1} & =\frac{\rho_{0}}{2X_{3,j,k}}e^{-X_{1,j,k}/k_{\rho }}X_{2,j,k}^{2}-g, \end{array}$$

(A34) $$\begin{array}{cc} c_{2} & =X_{2,j,k}+\frac{h}{2}q_{1}, \end{array}$$

(A35) $$\begin{array}{cc} q_{2} & =\frac{\rho_{0}}{2X_{3,j,k}}e^{-\left(X_{1,j,k}+hc_{1}/2\right)/k_{\rho }}{\left(X_{2,j,k}+\frac{h}{2}q_{1}\right)}^{2}-g, \end{array}$$

(A36) $$\begin{array}{cc} c_{3} & =X_{2,j,k}+\frac{h}{2}q_{2}, \end{array}$$

(A37) $$\begin{array}{cc} q_{3} & =\frac{\rho_{0}}{2X_{3,j,k}}e^{-\left(X_{1,j,k}+hc_{2}/2\right)/k_{\rho }}{\left(X_{2,j,k}+\frac{h}{2}q_{2}\right)}^{2}-g, \end{array}$$

(A38) $$\begin{array}{cc} c_{4} & =X_{2,j,k}+hq_{3}, \end{array}$$

(A39) $$\begin{array}{cc} q_{4} & =\frac{\rho_{0}}{2X_{3,j,k}}e^{-\left(X_{1,j,k}+hc_{3}/2\right)/k_{\rho }}{\left(X_{2,j,k}+\frac{h}{2}q_{3}\right)}^{2}-g. \end{array}$$

Using these coefficients, the sigma point time-update equations are given as follows:

(A40) $$\begin{array}{cc} X_{1,j,k+1}^{-} & =X_{1,j,k}+\frac{h}{6}\left(c_{1}+2c_{2}+2c_{3}+c_{4}\right), \end{array}$$

(A41) $$\begin{array}{cc} X_{2,j,k+1}^{-} & =X_{2,j,k}+\frac{h}{6}\left(q_{1}+2q_{2}+2q_{3}+q_{4}\right), \end{array}$$

(A42) $$\begin{array}{cc} X_{3,j,k+1}^{-} & =X_{3,j,k}, \end{array}$$

where j=1,…,7.

### Appendix C.3. Time-Update Equations for AB-UKF (Falling Body)

From Equations (64)–(66), the nonlinear dynamics $f_{c}\left(x\right)$ of the continuous-time state equation are redefined as follows:

(A43) $$\frac{d}{dt}\left[\begin{array}{c} x_{1}\\ x_{2}\\ x_{3} \end{array}\right]=\left[\begin{array}{c} f_{1}\\ f_{2}\\ f_{3} \end{array}\right]=\left[\begin{array}{c} x_{2}\\ \frac{\rho_{0}}{2x_{3}}e^{-x_{1}/k_{\rho }}x_{2}^{2}-g\\ 0 \end{array}\right]≜f_{c}\left(x\right).$$

The sigma point matrix $X_{k}$ at time *k* and the matrix $F_{c}\left(X_{k}\right)$ obtained by substituting $X_{k}$ into $f_{c}\left(x\right)$ are represented by the following 3×7 matrices:

(A44) $$\begin{array}{cc} X_{k} & =\left[\begin{array}{ccc} X_{1,1,k} & \ldots & X_{1,7,k}\\ X_{2,1,k} & \ldots & X_{2,7,k}\\ X_{3,1,k} & \ldots & X_{3,7,k} \end{array}\right], \end{array}$$

(A45) $$\begin{array}{cc} F_{c}\left(X_{k}\right) & =\left[\begin{array}{ccc} X_{2,1,k} & \ldots & X_{2,7,k}\\ \frac{\rho_{0}}{2X_{3,1,k}}e^{-X_{1,1,k}/k_{\rho }}X_{2,1,k}^{2}-g & \ldots & \frac{\rho_{0}}{2X_{3,7,k}}e^{-X_{1,7,k}/k_{\rho }}X_{2,7,k}^{2}-g\\ 0 & \ldots & 0 \end{array}\right]. \end{array}$$

To distinguish this $F_{c}\left(X_{k}\right)$ specific to the falling body model from the general form in Equation (9), we redefine it as $F_{c}^{fb}\left(X_{k}\right):=F_{c}\left(X_{k}\right)$. Then, the sigma point update equation using the fourth-order Adams–Bashforth method is given as follows:

(A46) $$\begin{array}{c} X_{k+1}^{-}=X_{k}+\frac{h}{24}\left(55F_{c}^{fb}\left(X_{k}\right)-59F_{c}^{fb}\left(X_{k-1}\right)+37F_{c}^{fb}\left(X_{k-2}\right)-9F_{c}^{fb}\left(X_{k-3}\right)\right). \end{array}$$

Here, the superscript fb denotes the falling body model.

## Appendix D. Details of Time-Update Equations for the UAV Model

This appendix presents the specific sigma point time-update equations for each method outlined in Section 7.2.

### Appendix D.1. Time-Update Equations for Euler-UKF (UAV)

Applying the Euler method to Equation (75) yields the following discrete-time state equation:

(A47) $$\begin{array}{c} \left[\begin{array}{c} x_{1,k+1}\\ x_{2,k+1}\\ \vdots \\ x_{11,k+1}\\ x_{12,k+1} \end{array}\right]=\left[\begin{array}{c} x_{1}+hx_{2}\\ x_{2}+h\left(A_{1}\left(x\right)T_{x}+A_{2}\left(x\right)T_{y}+A_{3}\left(x\right)T_{z}\right)\\ x_{3}+hx_{4}\\ x_{4}+h\left(A_{4}\left(x\right)T_{x}+A_{5}\left(x\right)T_{y}+A_{6}\left(x\right)T_{z}\right)\\ x_{5}+hx_{6}\\ x_{6}+h\left(A_{7}\left(x\right)T_{x}+A_{8}\left(x\right)T_{y}+A_{9}\left(x\right)T_{z}+g\right)\\ x_{7}+hx_{8}\\ x_{8}+h\left(\frac{1}{I_{Bxx}}\left(\tau_{x}^{B}+\tau_{x}^{c}\right)+x_{10}x_{12}\left(\frac{I_{Byy}-I_{Bzz}}{I_{Bxx}}\right)\right)\\ x_{9}+hx_{10}\\ x_{10}+h\left(\frac{1}{I_{Byy}}\left(\tau_{y}^{B}+\tau_{y}^{c}\right)+x_{8}x_{12}\left(\frac{I_{Bzz}-I_{Bxx}}{I_{Byy}}\right)\right)\\ x_{11}+hx_{12}\\ x_{12}+h\left(\frac{1}{I_{Bzz}}\left(\tau_{z}^{B}+\tau_{z}^{c}\right)+x_{8}x_{10}\left(\frac{I_{Bxx}-I_{Byy}}{I_{Bzz}}\right)\right) \end{array}\right]. \end{array}$$

Therefore, by substituting $X_{k}$ into Equation (A47), the time-update equation for the sigma points is derived as follows:

(A48) $$\begin{array}{cc} X_{1,j,k+1}^{-} & =X_{1,j,k}+hX_{2,j,k}, \end{array}$$

(A49) $$\begin{array}{cc} X_{2,j,k+1}^{-} & =X_{2,j,k}+h\left(A_{1}\left(X_{(9,11),j,k}\right)T_{x}+A_{2}\left(X_{(7,9,11),j,k}\right)T_{y}+A_{3}\left(X_{(7,9,11),j,k}\right)T_{z}\right), \end{array}$$

(A50) $$\begin{array}{cc} X_{3,j,k+1}^{-} & =X_{3,j,k}+hX_{4,j,k}, \end{array}$$

(A51) $$\begin{array}{cc} X_{4,j,k+1}^{-} & =X_{4,j,k}+h\left(A_{4}\left(X_{(9,11),j,k}\right)T_{x}+A_{5}\left(X_{(7,9,11),j,k}\right)T_{y}+A_{6}\left(X_{(7,9,11),j,k}\right)T_{z}\right), \end{array}$$

(A52) $$\begin{array}{cc} X_{5,j,k+1}^{-} & =X_{5,j,k}+hX_{6,j,k}, \end{array}$$

(A53) $$\begin{array}{cc} X_{6,j,k+1}^{-} & =X_{6,j,k}+h\left(A_{7}\left(X_{9,j,k}\right)T_{x}+A_{8}\left(X_{(7,9),j,k}\right)T_{y}+A_{9}\left(X_{(7,9),j,k}\right)T_{z}+g\right), \end{array}$$

(A54) $$\begin{array}{cc} X_{7,j,k+1}^{-} & =X_{7,j,k}+hX_{8,j,k}, \end{array}$$

(A55) $$\begin{array}{cc} X_{8,j,k+1}^{-} & =X_{8,j,k}+h\left(\frac{1}{I_{Bxx}}\left(\tau_{x}^{B}+\tau_{x}^{c}\right)+X_{10,j,k}X_{12,j,k}\left(\frac{I_{Byy}-I_{Bzz}}{I_{Bxx}}\right)\right), \end{array}$$

(A56) $$\begin{array}{cc} X_{9,j,k+1}^{-} & =X_{9,j,k}+hX_{10,j,k}, \end{array}$$

(A57) $$\begin{array}{cc} X_{10,j,k+1}^{-} & =X_{10,j,k}+h\left(\frac{1}{I_{Byy}}\left(\tau_{y}^{B}+\tau_{y}^{c}\right)+X_{8,j,k}X_{12,j,k}\left(\frac{I_{Bzz}-I_{Bxx}}{I_{Byy}}\right)\right), \end{array}$$

(A58) $$\begin{array}{cc} X_{11,j,k+1}^{-} & =X_{11,j,k}+hX_{12,j,k}, \end{array}$$

(A59) $$\begin{array}{cc} X_{12,j,k+1}^{-} & =X_{12,j,k}+h\left(\frac{1}{I_{Bzz}}\left(\tau_{z}^{B}+\tau_{z}^{c}\right)+X_{8,j,k}X_{10,j,k}\left(\frac{I_{Bxx}-I_{Byy}}{I_{Bzz}}\right)\right). \end{array}$$

Here, j=1,…,25.

### Appendix D.2. Time-Update Equations for RK-UKF (UAV)

To apply the fourth-order Runge–Kutta method to Equation (75), we define the derivatives $a_{1}\left(x\right)$–$a_{4}\left(x\right)$, …, $l_{1}\left(x\right)$–$l_{4}\left(x\right)$ for each state variable. Due to space limitations, it is not possible to list all equations, but representative ones are shown below:

(A60) $$\begin{array}{cc} a_{1}\left(x\right) & =f_{1}\left(x_{2}\right), \end{array}$$

(A61) $$\begin{array}{cc} a_{2}\left(x\right) & =f_{1}\left(x_{2}+\frac{b_{1}}{2}\right), \end{array}$$

(A62) $$\begin{array}{cc} a_{3}\left(x\right) & =f_{1}\left(x_{2}+\frac{b_{2}}{2}\right), \end{array}$$

(A63) $$\begin{array}{cc} a_{4}\left(x\right) & =f_{1}\left(x_{2}+b_{3}\right), \end{array}$$

(A64) $$\begin{array}{cc} \; & \vdots \end{array}$$

(A65) $$\begin{array}{cc} l_{1}\left(x\right) & =f_{12}\left(x_{8},x_{10}\right), \end{array}$$

(A66) $$\begin{array}{cc} l_{2}\left(x\right) & =f_{12}\left(x_{8}+\frac{h_{1}}{2},x_{10}+\frac{j_{1}}{2}\right), \end{array}$$

(A67) $$\begin{array}{cc} l_{3}\left(x\right) & =f_{12}\left(x_{8}+\frac{h_{2}}{2},x_{10}+\frac{j_{2}}{2}\right), \end{array}$$

(A68) $$\begin{array}{cc} l_{4}\left(x\right) & =f_{12}\left(x_{8}+h_{3},x_{10}+j_{3}\right). \end{array}$$

Using these derivatives, the time-update equations for the sigma points are derived as follows:

(A69) $$\begin{array}{cc} X_{1,j,k+1}^{-} & =X_{1,j,k}+\frac{h}{6}\left(a_{1}\left(X_{2,j,k}\right)+2a_{2}\left(X_{2,j,k}\right)+2a_{3}\left(X_{2,j,k}\right)+a_{4}\left(X_{2,j,k}\right)\right), \end{array}$$

(A70) $$\begin{array}{cc} X_{2,j,k+1}^{-} & =X_{2,j,k}+\frac{h}{6}\left(b_{1}\left(X_{(7,9,11),j,k}\right)+2b_{2}\left(X_{(7,9,11),j,k}\right)+2b_{3}\left(X_{(7,9,11),j,k}\right)+b_{4}\left(X_{(7,9,11),j,k}\right)\right), \end{array}$$

(A71) $$\begin{array}{cc} \; & \vdots \end{array}$$

(A72) $$\begin{array}{cc} X_{12,j,k+1}^{-} & =X_{12,j,k}+\frac{h}{6}\left(l_{1}\left(X_{(8,10),j,k}\right)+2l_{2}\left(X_{(8,10),j,k}\right)+2l_{3}\left(X_{(8,10),j,k}\right)+l_{4}\left(X_{(8,10),j,k}\right)\right). \end{array}$$

### Appendix D.3. Time-Update Equations for AB-UKF (UAV)

The sigma point matrix $X_{k}$ at time *k* and the matrix $F_{c}\left(X_{k}\right)$ obtained by substituting $X_{k}$ into Equation (75) are represented by the following 12×25 matrices:

(A73) $$\begin{array}{cc} X_{k} & =\left[\begin{array}{ccc} X_{1,1,k} & \ldots & X_{1,25,k}\\ \vdots & \ddots & \vdots \\ X_{12,1,k} & \ldots & X_{12,25,k} \end{array}\right], \end{array}$$

(A74) $$\begin{array}{cc} F_{c}\left(X_{k}\right) & =\left[\begin{array}{ccc} f_{1}\left(X_{1,1,k}\right) & \ldots & f_{1}\left(X_{1,25,k}\right)\\ \vdots & \ddots & \vdots \\ f_{12}\left(X_{12,1,k}\right) & \ldots & f_{12}\left(X_{12,25,k}\right) \end{array}\right]. \end{array}$$

To distinguish this $F_{c}\left(X_{k}\right)$ specific to the UAV model from the general form in Equation (9), we redefine it as $F_{c}^{U}\left(X_{k}\right):=F_{c}\left(X_{k}\right)$. Then, the sigma point update equations using the second- to sixth-order Adams–Bashforth methods are given as follows:

**2nd-order Adams–Bashforth method**:

(A75) $$\begin{array}{c} X_{k+1}^{-}=X_{k}+\frac{h}{2}\left(3F_{c}^{U}\left(X_{k}\right)-F_{c}^{U}\left(X_{k-1}\right)\right). \end{array}$$

**3rd-order Adams–Bashforth method**:

(A76) $$\begin{array}{c} X_{k+1}^{-}=X_{k}+\frac{h}{12}\left(23F_{c}^{U}\left(X_{k}\right)-16F_{c}^{U}\left(X_{k-1}\right)+5F_{c}^{U}\left(X_{k-2}\right)\right). \end{array}$$

**4th-order Adams–Bashforth method**:

(A77) $$\begin{array}{c} X_{k+1}^{-}=X_{k}+\frac{h}{24}\left(55F_{c}^{U}\left(X_{k}\right)-59F_{c}^{U}\left(X_{k-1}\right)+37F_{c}^{U}\left(X_{k-2}\right)-9F_{c}^{U}\left(X_{k-3}\right)\right). \end{array}$$

**5th-order Adams–Bashforth method**:

(A78) $$\begin{array}{cc} X_{k+1}^{-}=X_{k}+\frac{h}{720}( & 1901F_{c}^{U}\left(X_{k}\right)-2774F_{c}^{U}\left(X_{k-1}\right)+2616F_{c}^{U}\left(X_{k-2}\right)\\ \; & -1274F_{c}^{U}\left(X_{k-3}\right)+251F_{c}^{U}\left(X_{k-4}\right)). \end{array}$$

**6th-order Adams–Bashforth method**:

(A79) $$\begin{array}{cc} X_{k+1}^{-}=X_{k}+\frac{h}{1440}( & 4277F_{c}^{U}\left(X_{k}\right)-7923F_{c}^{U}\left(X_{k-1}\right)+9982F_{c}^{U}\left(X_{k-2}\right)\\ \; & -7298F_{c}^{U}\left(X_{k-3}\right)+2877F_{c}^{U}\left(X_{k-4}\right)-475F_{c}^{U}\left(X_{k-5}\right)). \end{array}$$
